# Synergy between B cell receptor/antigen uptake and MHCII peptide editing relies on HLA-DO tuning

**DOI:** 10.1038/s41598-019-50455-y

**Published:** 2019-09-25

**Authors:** Wei Jiang, Lital N. Adler, Henriette Macmillan, Elizabeth D. Mellins

**Affiliations:** 1 Department of Pediatrics – Human Gene Therapy, Stanford University School of medicine, Stanford, CA 94305 USA; 2 Stanford Immunology, Stanford University School of Medicine, Stanford, CA 94305 USA; 3 Present Address: Weizmann Institute of Science, Rehovot, Area Israel; 4 Present Address: Department of Medicine, University of California San Francisco, San Francisco, CA 94143 USA

**Keywords:** Antigen-presenting cells, MHC class II

## Abstract

B cell receptors and surface-displayed peptide/MHCII complexes constitute two key components of the B-cell machinery to sense signals and communicate with other cell types during antigen-triggered activation. However, critical pathways synergizing antigen-BCR interaction and antigenic peptide-MHCII presentation remain elusive. Here, we report the discovery of factors involved in establishing such synergy. We applied a single-cell measure coupled with super-resolution microscopy to investigate the integrated function of two lysosomal regulators for peptide loading, HLA-DM and HLA-DO. In model cell lines and human tonsillar B cells, we found that tunable DM/DO stoichiometry governs DM_free_ activity for exchange of placeholder CLIP peptides with high affinity MHCII ligands. Compared to their naïve counterparts, memory B cells with less DM_free_ concentrate a higher proportion of CLIP/MHCII in lysosomal compartments. Upon activation mediated by high affinity BCR, DO tuning is synchronized with antigen internalization and rapidly potentiates DM_free_ activity to optimize antigen presentation for T-cell recruitment.

## Introduction

When B cells encounter antigen, two steps adopting affinity checks for ligand-receptor interaction facilitate the mounting of an effective humoral response. In Step 1, antigen uptake and B cell signaling by B cell receptors (BCRs) relies on BCR affinity/avidity. In Step 2, peptides are selected for presentation to CD4 T cells, based on affinity of binding to major histocompatibility complex class II (MHCII) proteins^1^. Linking these two affinity checkpoints is fundamental to T-B interaction during the process needed to form germinal centers (GCs) and during the time B cells proliferate and differentiate within GC^2,3^. However, the pathways and events critical to creating this bridge are not established.

It is known that several key regulators within the MHCII peptide loading machinery modulate the effectiveness of specific peptide presentation and thus are likely involved in such bridging. Nascent MHCII associate with the invariant chain^4^, which limits the loading of ligands in the endoplasmic reticulum and directs MHCII to endosomal compartments, where Ii is degraded, leaving a nested set of peptides (CLIP) in the grooves of MHCII molecules. The non-classical MHC human leucocyte antigen HLA-DM (DM), a central Step 2 regulator, transiently associates with MHCII^5–7^ and selects MHCII ligands in a process called DM editing^8,9^. One critical function of DM editing is to facilitate the removal of placeholder CLIP peptides to allow the loading of high affinity MHCII ligands^10–13^.

In resting B cells, DM action is negatively modulated by another MHCII-encoded regulator, HLA-DO (DO)^14–16^. DO is also detectable in medullary thymic epithelial cells and certain dendritic cells (DCs)^1,17^. However, B cells are uniquely specialized antigen presenting cells (APCs) in which downregulation of DO is associated with efficiency of entrance into GC, where affinity maturation of BCRs and differentiation of B cells take place^18–21^. Further, memory B cells restore significantly high levels of DO after initial antigen exposure, affinity maturation and selection^18,19,21^. These observations suggest that understanding the maturation/activation-dependent tuning of DO levels will be key to uncovering a connection between the two affinity checkpoints. Structural evidence argues that DO mimics classical MHCII to bind DM, blocking the DM active site for MHCII interaction^22–24^. Therefore, lowering DO levels likely promotes DM editing; however, the concomitant decline of DM levels^19,21^ complicates understanding the functional role of DO tuning. Thus, to investigate this critical regulation requires an approach that integrates DM/DO function.

Here, we integrated the functions of DM and DO, using a novel single-cell parameter together with essential input from super-resolution microscopy. We found that the expression of DO in resting B cells is central in controlling DM/DO stoichiometry, whose nuanced variations between individual cells significantly influence the efficiency of CLIP removal from MHCII. By tuning the levels of DO to regulate DM/DO stoichiometry, a B cell can quickly establish synergy between the internalization of antigen bound by high affinity BCR and efficient selection for high affinity MHCII ligands. Our analysis of human tonsillar B cells reveals the importance of establishing tunable DM/DO stoichiometry during memory B cell development. Importantly, we discovered the 2-synchronized-affinity-checkpoint mechanism underlying the synergy.

## Results

### Lowering DO promotes increases in DM_free_/CLIP_freq_

**Figure 1:**
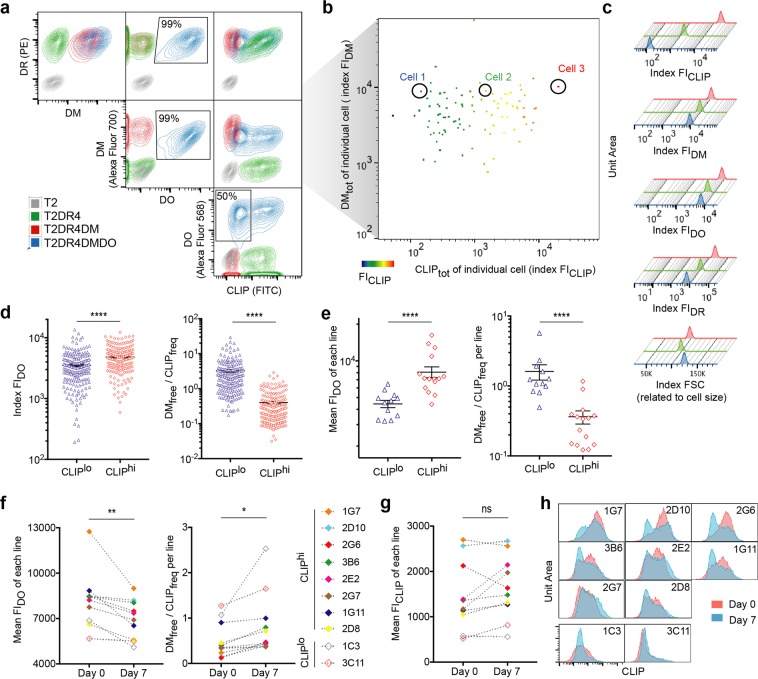
DM_free_/CLIP_freq_ measured in single cells is correlated with DO. (**a**) Contour plots of flow cytometric analysis (n = 4) of fixed/permeabilized T2 derivatives co-stained with fluorophore-conjugated antibodies specific for DM, DO, DR or CLIP, as indicated. (**b**) iFACS analysis (n = 3) of fixed/permeabilized T2DR4DMDO cells co-stained as in (**a**). Heatmap colors scaled to the index FI_CLIP_ of individual cells. (**c**) Index FIs of 3 representative cells as circled in (**b**) represented by stagger-offset histograms. (**d**) Comparisons of Index FI_DO_ of individual T2DR4DMDO cells or DM_free_/CLIP_freq_ determined by iFACS for individual cells from CLIP^lo^ and CLIP^hi^ groups. Data include 384 single cells. (**e**) Comparisons of mean FI_DO_ from single clonal lines or DM_free_/CLIP_freq_ determined by MFIs using flow cytometry for each line (CLIP^lo^ vs CLIP^hi^). (**f**,**g**) Pairwise comparisons (paired *t*-test) of mean FI_DO_ or DM_free_/CLIP_freq_ or mean FI_CLIP_ of each line (indicated by colors) before (Day 0) and after (Day 7) downregulation of DO expression. (**h**) Overlay of histograms showing the CLIP levels of each line prior to (Day 0) or after (Day 7) downregulation of DO expression. ns: non-significant, p > 0.05; *p < 0.05, **p < 0.01, ****p < 0.0001.

The catalytic activity of DM results in kinetically enhanced exchange of MHCII-bound CLIP for antigenic peptides^11,12^. However, the influences of DO inhibition and intracellular trafficking of class II proteins on the dynamic peptide exchange process in cells^25–29^ make it challenging to correlate DM/DO function with CLIP levels. This is especially the case when DM and DO are concomitantly downregulated during B cell engagement with antigen^19,30^. As an initial step towards dissection of this correlation in a physiological setting, we first used a panel of related, simplified model cell lines^12^, generated by sequential transfection of MHCII^null^/DM^null^/DO^null^ TxB hybridoma T2 cells with the key molecules of interest: a single MHCII allele, HLA-DR4 (DRB1*04:01), then DM and then DO. CLIP peptides associated with DR4 were prominent in T2DR4 cells (DM^null^/DO^null^), whereas expression of DM in T2DR4DM cells (DO^null^) resulted in their near-complete removal, indicating sufficient catalysis by DM and available (extracelluar and endogenous) sources of high affinity peptides to replace CLIP (Fig. 1a). Consistent with the inhibitory role of DO on DM activity, the expression of DO in T2DR4DMDO cells significantly impaired CLIP removal. Unexpectedly, although over 99% of T2DR4DMDO cells expressed comparable levels of DM/DO, half of these DO^+^ cells were unable to limit DM catalysis of CLIP removal (Fig. 1a).

To investigate inter-cell differences in DM/DO effects on CLIP removal, we evaluated T2DR4DMDO cells individually using index single-cell fluorescence-activated cell sorting (iFACS). Representative single cells with significantly different levels of total CLIP (CLIP_tot_) showed almost identical levels of DM_tot_, measured as the index fluorescence intensity (FI_DM_) (Fig. 1b). Differences in DR_tot_ or DO_tot_ among these cells were also not significant (Fig. 1c), unless we compared the average index FI by dividing hundreds of single cells into CLIP^lo^ and CLIP^hi^ groups (Supplementary Fig. 1a). Importantly, we found that a new parameter DM_free_/CLIP_freq_ revealed the influence of DM/DO stoichiometry on CLIP removal. DM_free_ (=DM_tot_ − DM_DO-associated_ = FI_DM_/FSC − FI_DO_/FSC) represents the density of free DM, where DO-associated DM was estimated by DO_tot_, as almost all DO *in vivo* is associated with DM^25,31,32^. CLIP_freq_ (=CLIP_tot_/DR_tot_ = FI_CLIP_/FI_DR_) reflects the frequency of CLIP-loaded DR (designated by “CLIP” hereafter). We found that DM_free_/CLIP_freq_ was inversely related to DO_tot_ level and reflected the efficacy of DM editing (Fig. 1d).

As DM_free_/CLIP_freq_ is based on single-cell measurements, we generated clonal lines expanded from individually sorted single T2DR4DMDO cells (Supplementary Fig. 1b) and tested the feasibility of using DM_free_/CLIP_freq_ to reveal the correlation between DM/DO regulation and CLIP release. We calculated DM_free_/CLIP_freq_ per line based on the mean FI of each protein of each clonal line (Supplementary Fig. 1c). Similar to the iFACS results with single cells, clonal lines isolated from the CLIP^lo^ group with lower levels of mean FI_DO_ had higher DM_free_/CLIP_freq_ per line than those from the CLIP^hi^ group (Fig. 1e). We also cultured 10 single clonal lines under no selection pressure for DO expression for a week and tested the associated outcome of DO downregulation indicated by DM_free_/CLIP_freq_. The decrease of DO expression due to a lack of selection resulted in an increase of DM_free_/CLIP_freq_ per line (Fig. 1f). A similar inverse correlation was also observed when another 6 single clonal lines were cultured with selection for increased DO expression (Supplementary Fig. 1d). Unexpectedly, the mean FI_CLIP_ per line was not coordinately decreased when the mean FI_DO_ of the corresponding clonal line diminished (Fig. 1g), due to the emerging heterogeneity in cells expanded from single clones (Fig. 1h; Supplementary Fig. 1e). This result implies the necessity of single-cell measurements in the study of DM/DO function even when the variation of DM/DO is as small as within a single clonal line, and indicates DM_free_/CLIP_freq_ rather than the actual CLIP level^14,33,34^ as an efficient measure of the physiological outcome of DM/DO regulation.

### DO regulation tunes DM_free_ to facilitate CLIP_int_ release and limit CLIP_srf_

**Figure 2:**
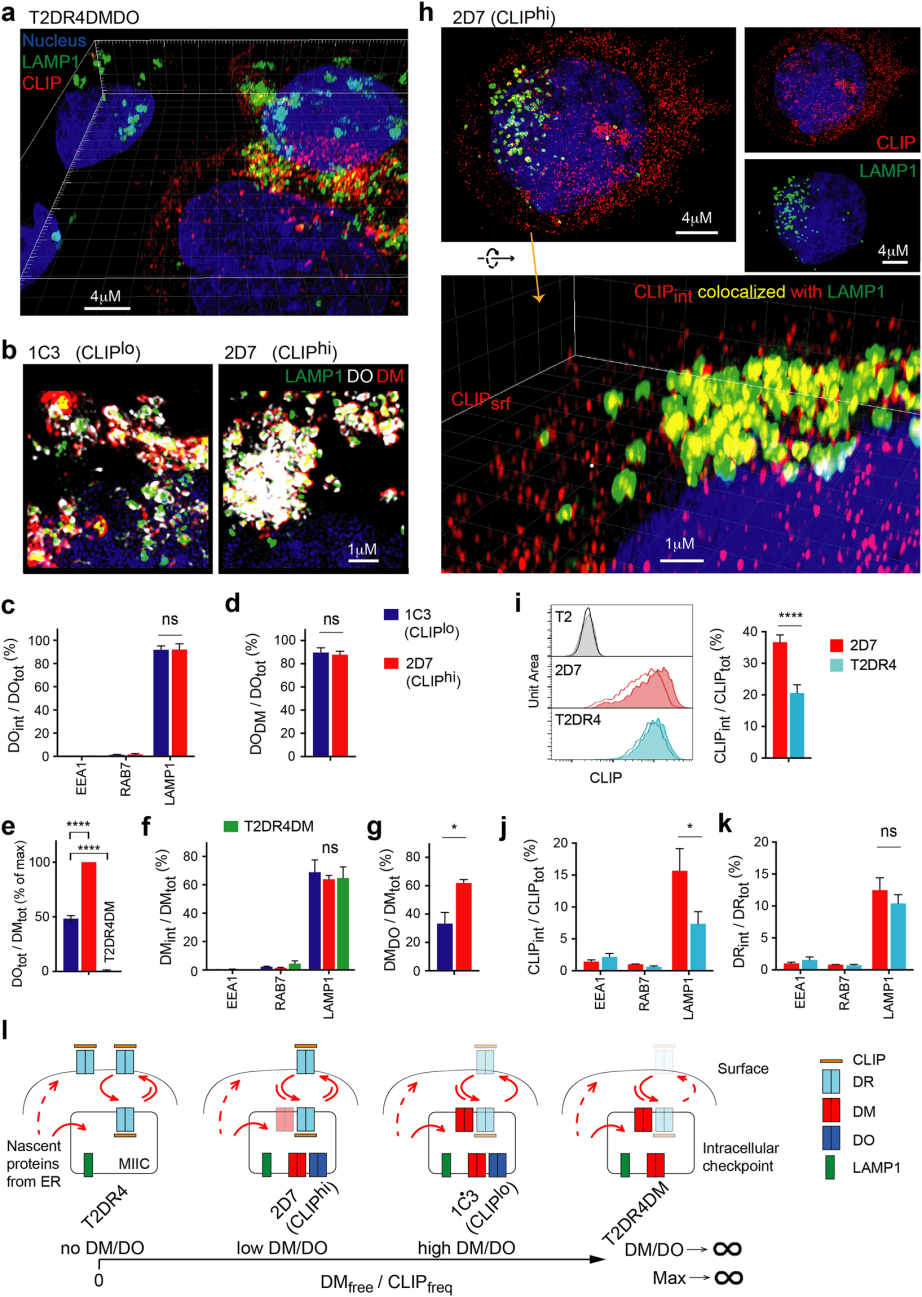
DO tunes DM_free_/CLIP_freq_ in LAMP1^+^ compartments despite surface display of CLIP. (**a**) Representative 3D-SIM overlay views of fixed/permeabilized T2DR4DMDO cells co-stained for LAMP1 and CLIP. Cells analyzed (N_cell_): 18 CLIP^hi^ and 19 CLIP^lo^ cells in 10 reconstructed 3D images. Experimental replicates (n) = 3. (**b**) Representative 3D-SIM overlay views of fixed/permeabilized 1C3 or 2D7 cells co-stained for LAMP1, DM and DO (see also Supplementary Fig. 2). n = 3. (**c**) Comparison of %DO co-localized with the indicated endosomal marker in 1C3 and 2D7 cells. N_cell_ (from left to right): 13, 8, 16, 15, 14, 16. (**d**) Comparison of %DO co-localized with DM in 1C3 and 2D7 cells. N_cell_: 25, 21. (**e**) Flow cytometric analysis (n = 4) of fixed/permeabilized 1C3, 2D7, or T2DR4DM cells co-stained for DM and DO. The ratio of mean FI_DO_ to mean FI_DM_ for each cell line was normalized to that for 2D7 cells. (**f**) Comparison of %DM co-localized with the indicated marker in 1C3 and 2D7 cells. N_cell_: 13, 8, 4, 12, 18, 21, 14, 18, 14. (**g**) Comparison %DM co-localized with DO in 1C3 and 2D7 cells. N_cell_: 25, 21. (**h**) 3D-SIM analysis (n = 3) of fixed/permeabilized 1C3 or 2D7 co-stained for LAMP1 and CLIP. Shown are overlay, single channel, and rotated zoom-in views. (**i**) Flow cytometric analysis (n = 3) of CLIP_srf_ (line) and CLIP_tot_ (filled) in T2, 2D7 or T2DR4 cells. MFI of CLIP_int_ = MFI of CLIP_tot_ – MFI of CLIP_srf_. (**j**,**k**) Comparisons of %CLIP or %DR co-localized with the indicated marker in 2D7 and T2DR4 cells (see also Supplementary Fig. 3). N_cell_ (same for **j**,**k**): 10, 11, 4, 7, 30, 37. (**l**) The functional correlation between varied levels of DM/DO and their regulation of CLIP removal. Transparency is related to protein levels per cell. Data are represented as mean ± SEM. ns: non-significant, p > 0.05; *p < 0.05, ****p < 0.0001.

Consistent with flow cytometric studies, three-dimensional structured illumination microscopy (3D-SIM) detected very low levels of CLIP in almost half of T2DR4DMDO cells (Fig. 2a). 3D-SIM also showed high levels of surface CLIP (CLIP_srf_) peptides on CLIP^hi^ cells. As DM primarily resides in the intracellular MHCII-containing compartments (MIIC)^25,26^, the engagement of CLIP_srf_ by DM becomes spatially limited. This raised a question as to whether there was any difference in protein localization in CLIP^lo^ and CLIP^hi^ cells that affected CLIP removal by DM_free_.

To address this question, which is essential to the understanding of the physiological meaning of DM_free_/CLIP_freq_, we compared localization of DM and DO in two representative single clonal lines 1C3 (CLIP^lo^) and 2D7 (CLIP^hi^), each of which had minimum clonal variation in cell cultures with appropriate selection for DO expression. 3D-SIM analysis of both 1C3 and 2D7 cells showed that, by staining, most DM and DO molecules were in close proximity (<200 nm apart) to lysosomal-associated membrane protein LAMP1 (Fig. 2b; Supplementary Movies 1,2), but not to early endosome antigen EEA1 or late endosome Ras-related protein RAB7 (Supplementary Fig. 2a). The surface-surface co-localization extension built in the Imaris v9.4 software (ImarisXT, Bitplane Inc, http://bitplane.com) enabled calculation of the percentage of DM and DO that co-localized (<80 nm in xy plane and <160 nm in z axis) with each other or with tested endosomal markers (Supplementary Fig. 2b). This super resolution measurement revealed that, in both 1C3 and 2D7 cells, the majority of DO co-localized with LAMP1 and DM (Fig. 2c,d), confirming the lysosomal destination of DO, whose intracellular trafficking requires DM association^25,31,32^. Varied DM/DO ratios in DO^+^ 1C3 and 2D7 or DO^null^ T2DR4DM cells (Fig. 2e) had minimal influence on the lysosomal localization of DM (Fig. 2f); however, a significantly lower percentage of DM co-localized with DO in 1C3 than in 2D7 cells (Fig. 2g). These results demonstrate unchanged localization of lysosomal DM_free_ in cells with varied levels of DM/DO, although in CLIP^hi^ clones, less DM_free_ contributed to the lower DM_free_/CLIP_freq_.

We also evaluated the distribution of CLIP in DM^+^/DO^+^ 2D7 vs DM^null^/DO^null^ T2DR4 cells to determine any differences between the two cell lines. Super-resolution SIM allowed separation of CLIP_srf_ and CLIP_int_, and further quantification of the percentage of CLIP peptides that co-localized with various endosomal markers (Fig. 2h; Supplementary Fig. 3 and Movies 3-5). Compared to T2DR4, 2D7 cells showed significantly higher percentage of intracellular CLIP (CLIP_int_; Fig. 2i), which is mostly co-localized with LAMP1 (Fig. 2j). We also noted the presence of slightly more DR co-localized with LAMP1 in 2D7 versus T2DR4 cells (Fig. 2k), but not to the degree that is likely to fully explain the increased proportion of CLIP_int_. Rather, increased CLIP_int_ percentage in lysosomal compartments is likely attributable to DM/DO expression in 2D7 cells. Indeed, the presence of slightly more DR co-localized with LAMP1 in 2D7 versus T2DR4 cells (Fig. 2k) also likely reflects a functional outcome of DM/DO expression, as the presence of these chaperones may enhance the ability of DR to survive lysosomal pH^35–37^ and/or increase its residence time in the compartment.

The study of model cell lines demonstrates that both DM/DO expression and their stoichiometry influence the efficacy of CLIP removal and the intracellular accumulation (residence time) of CLIP_LAMP1_. DM_free_/CLIP_freq_ is a sensitive measure for DM_free_ at the LAMP1^+^ intracellular checkpoint that catalyzes CLIP_int_ removal and limits CLIP_srf_ (Fig. 2l). Lowering DO promotes the increase of DM_free_/CLIP_freq_, which represents the effectiveness of DM editing.

### Memory B cells possess low DM_free_/CLIP_freq_ and high CLIP_LAMP1_/CLIP_tot_

**Figure 3:**
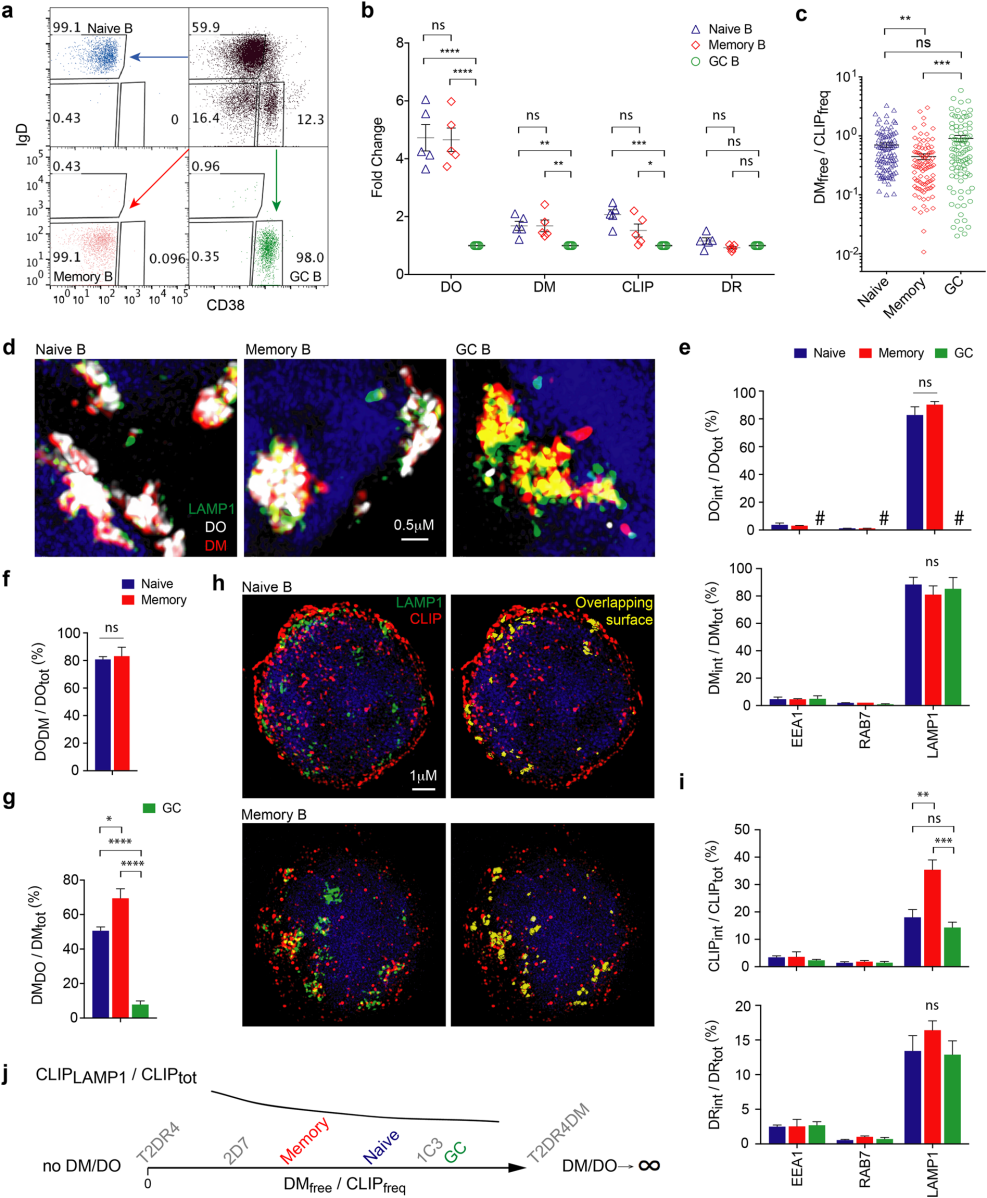
Low DM_free_/CLIP_freq_ and high CLIP_LAMP1_/CLIP_tot_ in memory B cells. (**a**) Naïve, memory and GC sub-populations sorted from human tonsil B cells and tested for purity. (**b**) Fixed/permeabilized tonsillar B cell subsets co-stained for DO, DM, DR, and CLIP, and analyzed by flow cytometry (n = 5). Mean FIs was normalized to that for GC B cells. (**c**) A comparison of DM_free_/CLIP_freq_ determined by iFACS of individual B cells from different sub-populations. Data at each condition include a 96-well plate of single cells from the same donor. Experiments were repeated with equivalent results using cells from three donors. (**d**) 3D-SIM analysis (n = 3) of fixed/permeabilized B cells co-stained for LAMP1, DM, and DO. (**e**) Comparisons of %DO or %DM co-localized with the indicated endosomal marker in different subtypes of B cells. ^#^Some GC B cells had almost no DO, although any detectable DO was co-localized with LAMP1 (see also Supplementary Fig. 4). N_cell_ (from left to right): 7, 13, 13, 21, 19, 15, 20, 14, 16. (**f**) %DO co-localized with DM in different subtypes of B cells. N_cell_: 25, 21. (**g**) %DM co-localized with DO in different subtypes of B cells. N_cell_: 52, 51, 69. (**h**) 3D-SIM analysis (n = 3) of fixed/permeabilized naïve or memory B cells co-stained for LAMP1 and CLIP. Right panel: an overlay view of the CLIP channel (red) and the calculated LAMP1/CLIP overlapping surface (yellow). (**i**) Comparisons of %CLIP or %DR co-localized with the indicated endosomal marker in different subtypes of B cells (see also Supplementary Fig. 5). N_cell_: 24, 21, 26, 19, 18, 16, 52, 60, 40. (**j**) An inverse correlation between CLIP_LAMP1_/CLIP_tot_ and DM_free_/CLIP_freq_ among tonsil B cell sub-populations. Data are represented as mean ± SEM. ns: non-significant, p > 0.05; *p < 0.05, **p < 0.01; ***p < 0.001; ****p < 0.0001.

We next applied DM_free_/CLIP_freq_ in the comparison of primary B cell sub-populations with varied levels of DM/DO. Human tonsillar B cells were sorted by FACS into three canonical subsets: naïve (IgD^+^/CD38^−/lo^), memory (IgD^−^/CD38^−/lo^), and GC (IgD^-^/CD38^hi^) (Fig. 3a; Supplementary Fig. 4a) as previously defined^18,19,21^. Bulk analysis of fluorescently labeled B cells by flow cytometry revealed the concomitant decrease of mean FIs representing levels of DO (most dramatically), DM and CLIP in GC B cells, whereas differences between naïve and memory cells were minimal (Fig. 3b; Supplementary Fig. 4b). Using iFACS to analyze individual cells, we found higher DM_free_/CLIP_freq_ on average in GC (Fig. 3c; Supplementary Fig. 4c), indicating higher efficacy of CLIP removal by DM catalysis, which is likely necessary for these cells to receive help from T_FH_ cells. In contrast, the significantly lower mean DM_free_/CLIP_freq_ in memory than the other two subtypes of B cells implied a higher demand for potentiation of DM_free_-catalyzed CLIP removal by memory B cells upon activation.

Like the localization analysis in model cell lines, we also determined the site(s) of DM_free_ in the tonsillar B cell subsets. We analyzed DM and DO by SIM and found DM was co-localized with LAMP1 (Fig. 3d; Supplementary Fig. 4d and Movies 6-8). The percentage of DM co-localized with various endosomal markers measured by 3D-SIM showed no difference between non-GC (naïve and memory) and activated GC B cells (Fig. 3e). Very similar to the results observed in T2 cell lines, the majority of DO co-localized with DM in non-GC DO^+^ B cell sub-populations (Fig. 3f). However, a significantly higher percentage of DM in memory than naive cells co-localized with DO (Fig. 3g), indicating that less DM_free_ at LAMP1 + compartments resulted in lower DM_free_/CLIP_freq_ in memory cells (Fig. 3c). In addition, the quantitative SIM analysis enabled further differentiation of memory from naive cells by CLIP_int_ distribution (Fig. 3h; Supplementary Fig. 5 and Movies 9,10). Memory cells with the lowest average DM_free_/CLIP_freq_ showed significantly higher percentage of CLIP (and slightly higher percentage of DR) co-localized with LAMP1 compared with naïve and GC B cells (Fig. 3i), despite indistinguishable levels of CLIP_tot_ or DR_tot_ between memory and naïve B cells (Fig. 3b).

Collectively, these results agree with the model suggested by the analysis of T2 cell lines and indicate that the net intracellular accumulation of CLIP (CLIP_LAMP1_/CLIP_tot_) inversely correlates with DM_free_/CLIP_freq_ in primary B cells (Fig. 3j). Non-GC B cells, especially memory (vs naïve) cells, with lower DM_free_/CLIP_freq_ and higher levels of CLIP-loaded MHCII in DM_free_^+^/LAMP1^+^ compartments, must experience stronger potentiation to efficiently replace CLIP with high affinity MHCII ligands during antigen engagement at pre-GC or GC phases.

### DO downregulation potentiates DM_free_ activity in memory B cells

**Figure 4:**
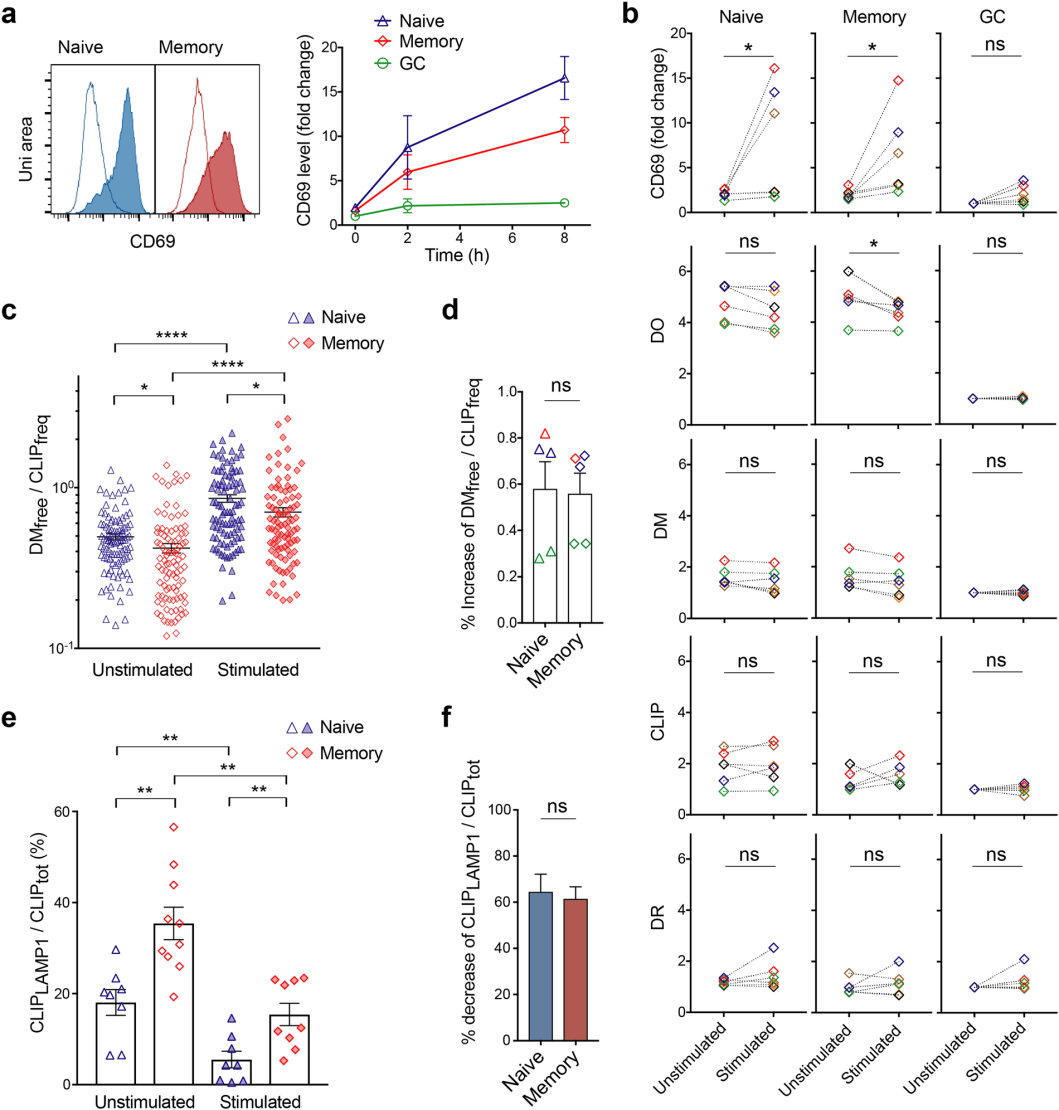
Similar changes of DM_free_/CLIP_freq_ and CLIP_LAMP1_/CLIP_tot_ in activated memory vs naive B cells rely on different extent of DO downregulation. (**a**) Flow cytometric analysis of stimulated (filled) or unstimulated (line) cells incubated with or without anti-IgG/anti-IgM Abs, CD40L, IL2, and IL4 for the indicated time and fixed/permeabilized before staining of total CD69. Right panel: MFI fold change of stimulated over unstimulated samples was normalized to that of GC B cells at time 0. n = 3. (**b**) Cells from 6 donors (indicated by colors) stimulated as in (**a**) for 2 h and compared with their unstimulated counterparts (paired *t*-test) for CD69, DO, DM, CLIP and DR using flow cytometric analysis. MFI normalized to that of unstimulated GC B cells. (**c**) Comparisons of DM_free_/CLIP_freq_ determined by iFACS of individual naïve and memory B cells that were unstimulated or stimulated as in (**a**) for 2 h. Data at each condition include a 96-well plate of single B cells from the same donor. Experiments were repeated 5 times using cells from three donors. (**d**) The %increase of average DM_free_/CLIP_freq_ per replication (open symbol) from unstimulated to stimulated naïve vs memory B cells from three donors (indicated by colors) as mentioned in (**c**). (**e**) Comparisons of %CLIP co-localized with LAMP1 (CLIP_LAMP1_/CLIP_tot_) quantified by 3D-SIM for unstimulated or stimulated naïve vs memory B cells. N_cell_: 52, 60, 47, 39. (**f**) The %decrease of average CLIP_LAMP1_/CLIP_tot_ per experiment (n = 3) from unstimulated to stimulated naïve vs memory B cells. Data are represented as mean ± SEM. ns: non-significant, p > 0.05; *p < 0.05, **p < 0.01; ****p < 0.0001.

To evaluate the potentiation of effective editing by active DM_free_ in non-GC B cells, we sought to activate both naïve and memory cells *in vitro*. After BCR engagement with antigen and T_FH_ cell-B cell interaction at the T/B border, GCs are formed within 24 h^3,38^, indicating that antigen processing and presentation involving DO downregulation occur within hours, as observed previously^30^. In a scheme that ensured *in vitro* activation of resting B cells mimicking a T-dependent pathway, we cross-linked BCR with anti-IgG and anti-IgM antibodies along with triggering CD40-mediated signaling, using recombinant CD40 ligand (CD40L)^39^. The total level of an early activation marker, CD69, in both non-GC (naïve and memory) but not GC B cells (already activated), significantly increased as early as 2 h post-activation (Fig. 4a). 6 h later, we observed increased levels of another activation marker, the co-stimulatory molecule, CD86 in both naïve and memory cells (Supplementary Fig. 6a), confirming successful *in vitro* activation.

We next compared naïve and memory B cells that had been cultured with/without stimuli for only 2 h, when there was negligible false positive auto-fluorescence associated with dead cells emerging (e.g., >4 h) from the *in vitro* culturing (Supplementary Fig. 6b). At 2 h, moderate and significant decreases of DO levels appeared in naïve and memory cells, respectively, whereas changes in levels of DM, CLIP or DR did not reach significance using bulk analysis (Fig. 4b). Using single-cell measurement, we found significantly increased DM_free_/CLIP_freq_ in both naïve and memory cells after stimulation (Fig. 4c), and the percentage of increase appeared to be similar in both types of B cells (Fig. 4d). In addition, CLIP co-localized with LAMP1 also significantly decreased by a similar percentage in both types of cells (Fig. 4e,f). The activation of naïve and memory cells via cross-linking of BCR drives similar % changes in DM_free_/CLIP_freq_ and CLIP_LAMP1_/CLIP_tot_, demonstrating the capability of potentiation for CLIP removal in both non-GC subsets. However, the more dramatic changes of DO levels in memory B cells (but not other related protein levels) confirmed their greater dependence on DO regulation to generate greater amount of active DM_free_ and to drive such potentiation. The difference between affinity matured memory cells and their naïve counterparts implies a critical role of DO downregulation during BCR-mediated activation.

### Prompt DO regulation is synchronized with high affinity BCR-antigen uptake

**Figure 5:**
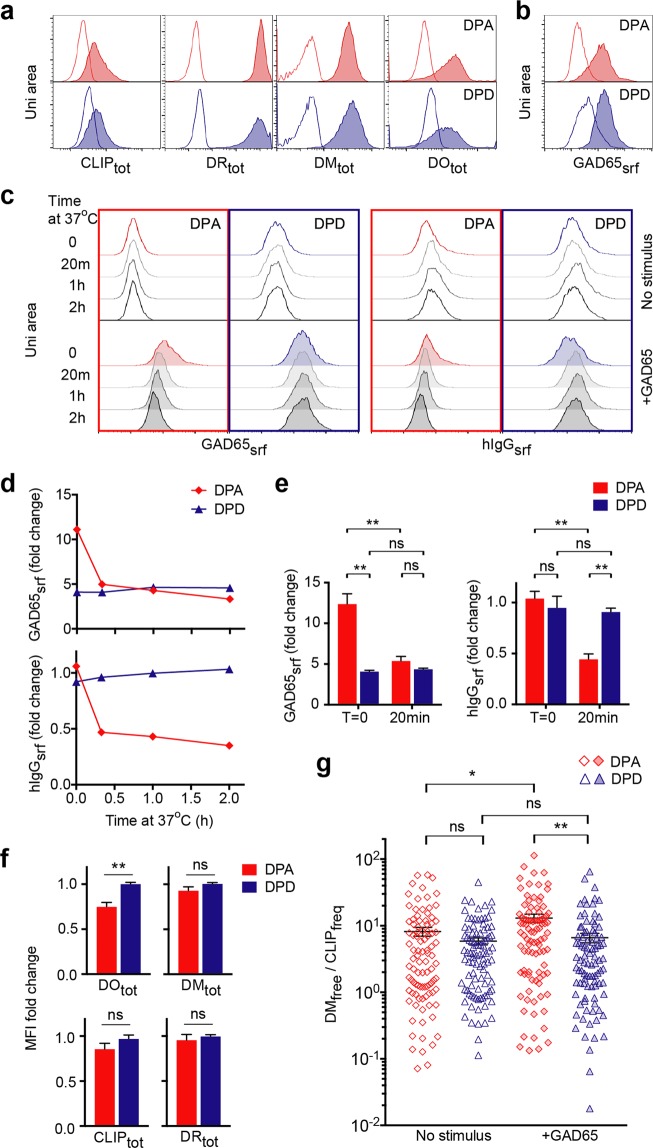
Increases of DM_free_/CLIP_freq_ in DPA attributed to fast GAD65-hIgG internalization and DO downregulation. (**a**) Flow cytometric analysis of fixed/permeabilized DPA or DPD cells co-stained for total CLIP, DR, DM, and DO. Lines indicate the corresponding unstained cells. n = 3. (**b**) DPA or DPD cells were incubated with GAD65 on ice followed by detection of surface GAD65 using mouse anti-GAD65 antibodies and analysis by flow cytometry. Lines indicate the corresponding cells without GAD65. n = 3. (**c**) GAD65-loaded (filled) or GAD65-unexposed (line) DPA (red) or DPD (blue) cells were incubated with (+GAD65) or without (no stimulus) GAD65 at 37 °C for the indicated time before detection of surface GAD65 using mouse anti-GAD65 antibodies or surface hIgG using mouse anti-hIgG antibodies and analysis by flow cytometry. n = 3. (**d**) Fast internalization of GAD65 and hIgG in DPA cells. Fold change indicates MFI at + GAD65 condition over no stimulus condition as shown in (**c**). (**e**) Comparisons of surface GAD65 or surface hIgG fold change between 0 and 20 min of incubation of DPA or DPD cells with GAD65 at 37 °C. n = 4. (**f**) Comparisons of fold change of total DO, DM, CLIP or DR in GAD65-loaded DPA and DPD cells that were further incubated with GAD65 at 37 °C for 20 min and fixed/permeabilized before flow cytometric analysis. 1 indicates no change. n = 5. (**g**) Comparisons of DM_free_/CLIP_freq_ determined by iFACS of individual DPA and DPD cells that were incubated without stimulus (no stimulus) or pre-loaded with GAD65 and then incubated with GAD65 ( + GAD65) at 37 °C for 20 min. n = 3. Data at each condition include a 96-well plate of single cells. Data are represented as mean ± SEM. ns: non-significant, p > 0.05; *p < 0.05, **p < 0.01.

To test whether there is any correlation between antigen-BCR and DO regulation, we examined the functional outcome during antigen-specific activation in human B cell lines, whose immunoglobulin G (IgG) BCRs have affinities that differ by over 2 orders of magnitude for the autoantigen glutamic acid decarboxylase (GAD65): DPA (<1 nM) and DPD (>100 nM)^40^. Flow cytometry detected DM, DO, DR and CLIP in both B cell lines (Fig. 5a).

In an assay where we tested the internalization of BCRs after GAD65-binding^41^, we first limited endocytosis by staining cells with GAD65 on ice and confirmed GAD65-binding on the surface (GAD65_srf_) of both cell lines (Fig. 5b). We then let cells incubate at 37 °C to allow internalization and measured the loss of GAD65_srf_ or surface human IgG (hIgG_srf_). There was a parallel, time-dependent decrease of GAD65_srf_ and hIgG_srf_ observed in DPA but not DPD cells (Fig. 5c). The internalization was fast, as the majority of protein loss on the surface of DPA cells occurred within 20 min (Fig. 5d). Notably, the background-subtracted GAD65_srf_ level in DPA cells decreased to a level similar to that of DPD cells (Fig. 5e), suggesting that there is a limit to the degree of internalization and internalization is BCR affinity-dependent. Of note, BCRs of both DPA and DPD cells are internalized after cross-linking by polyclonal antibodies, which overcomes BCR affinity differences (Supplementary Fig. 7a).

Within the same time frame of GAD65-hIgG internalization, we observed a significant decrease in the level of DO in DPA compared with DPD cells, whose DO level had negligible changes (Fig. 5f). Very importantly, this response in DPA cells was fast, occurring within 20 min, when the proliferation of cell lines had minimal contributions to protein expression. Consistent with this, we observed nearly unchanged levels of DM, CLIP or DR (Fig. 5f), or even CD69 (Supplementary Fig. 7b) in both cell lines. Similarly, DO levels decrease by 15%, within 20 min of the *in vitro* activation of human tonsil-derived resting B cells (particularly memory), while the level of CD69 is still unchanged (Supplementary Fig. 7c). Using single-cell measurements, we further found a significant increase of DM_free_/CLIP_freq_ in DPA but not in DPD cells (Fig. 5g), demonstrating a substantially boosted efficacy for DM_free_-catalyzed CLIP removal following the quick internalization of antigen-BCR complexes and downregulation of DO in cells that expressed appreciably higher affinity BCRs for the GAD65 antigen.

## Discussion

### Essential role of DM/DO stoichiometry

The requirement of DO downregulation for B cell activation and entry into GC for proliferation, affinity maturation and selection^18–21^ indicates highly regulated peptide loading machinery inside B cells to optimize T-B interaction for humoral responses. Studies using DO deletion or DO ectopic expression have shown different effects on the functional outcome of B cell presentation of various antigens^15,16,32,42,43^, including both autoimmune and anti-viral responses^44–46^. However, the physiological tuning of DO levels and associated changes in DM/DO stoichiometry is more nuanced than a process that alters the regulatory machinery either by completely removing DO or by switching on DO activity.

In this study, we developed a model system to compare B cell lines with a range of DM/DO ratios at steady state. At the single cell level, we were able to capture the functional outcomes of subtle changes in DM/DO stoichiometry and found that the presence of DO does not always completely block DM-catalyzed removal of MHCII-associated CLIP (e.g., cell line 1C3 with a decent level of DO has almost no CLIP). This unexpected finding provides evidence for the essential role of local DM/DO stoichiometry in the CLIP removal process. Importantly, the single cell measurements enabled the discovery of a key parameter, DM_free_/CLIP_freq_, which best illustrated this relationship both experimentally and theoretically (detailed below).

### Physiological relevance of DM_free_/CLIP_freq_ to the effectiveness of DM editing

**Figure 6:**
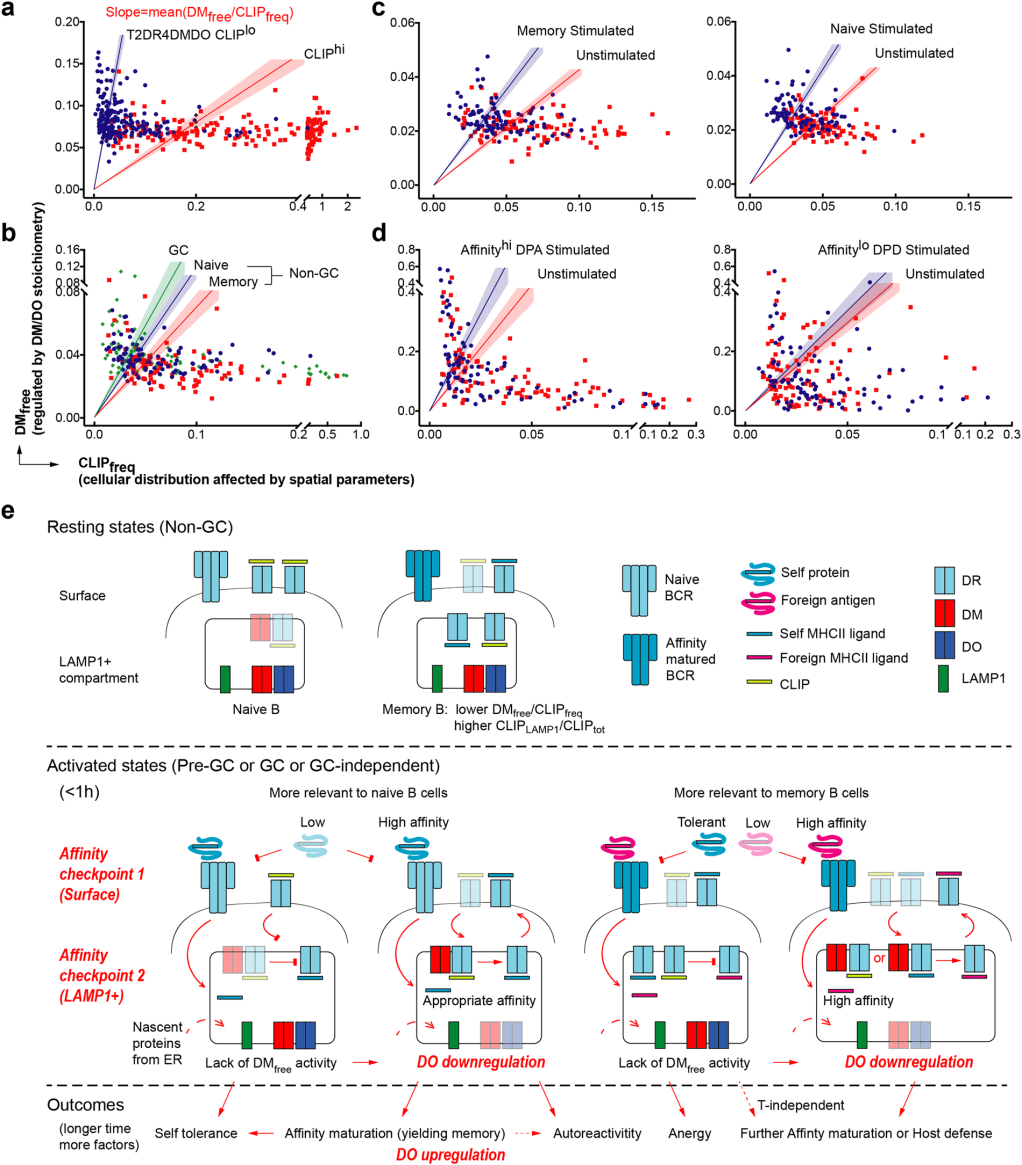
The molecular and cellular impact of fine-tuning DM/DO stoichiometry. (**a**–**d**) Mathematical and catalytic meaning of DM_free_/CLIP_freq_ illustrated by alternative presentation of data shown in Figs 1d, 3c, 4c and 5g, respectively. Each dot represents an individual cell analyzed by iFACS. Single cells from distinct groups are differentiated by colors, as indicated. The slope of a line connecting the origin with each dot equals to DM_free_/CLIP_freq_ in the cell. The line with shaded range indicates mean ± SEM of DM_free_/CLIP_freq_ for the group of cells using the same color-code. (**e**) Two synchronized affinity checkpoints for antigen presentation in B cells. Ligands with different binding affinities or proteins at different levels per cell are indicated using different degrees of color intensity.

The peptide loading of MHCII involves MHCII synthesis, antigen processing, complex regulations and catalytic reactions. The relevant proteins traffic throughout multiple intracellular compartments and shuttle between these compartments and the plasma membrane^1^. Our single cell analysis has taken various factors into account while focusing on elucidation of the impact of DM/DO expression and their stoichiometry on the efficacy of CLIP removal. Mathematically, DM_free_/CLIP_freq_ is the slope of a line connecting the origin with each dot that represents an individually analyzed cell on the DM_free_ vs CLIP_freq_ plot and a line can be drawn to represent the mean +/− SEM (Fig. 6a–d). Each comparison reveals the efficiency of active DM_free_, which scans peptide/MHCII complexes and catalyzes the exchange of bound CLIP (representative of low affinity peptides) for high affinity ligands: the higher DM_free_/CLIP_freq_ is, the more effective DM editing is. From an enzymatic perspective, a small amount of active DM_free_ molecules should catalyze the replacement of most CLIP (or low affinity) peptides given enough time. However, the varied effectiveness of DM editing, reflected by different DM_free_/CLIP_freq_ of different B cells, can be understood if one considers both temporal and spatial parameters in these cells. DM editing can reach a limit if the accumulation rate of CLIP surpasses the speed of DM-catalyzed CLIP removal. Reciprocally, this limit may increase the residence time of CLIP/MHCII in MIIC. This is in line with our observation that the 2D7 clonal line or non-GC B cells with low DM_free_/CLIP_freq_ maintain an appreciable proportion of CLIP/MHCII in LAMP1^+^ compartments (CLIP_LAMP1_/CLIP_tot_) at steady state.

The spatial parameter contributes to variations in DM_free_/CLIP_freq_ by mainly affecting the distribution of CLIP, as 3D-SIM analysis detected minimal DM/DO outside of LAMP1^+^ compartments at steady state and nearly all DO signals were in close proximity to DM signals. This supports previous conclusions that both DM and DO are lysosomal residents and DO requires chaperoning by DM^25,31,32^. Therefore, in contrast to some *in vitro* suggestions^47^, DO is unlikely to act separately from DM on peptide exchange *in vivo*. This perspective is consistent with the requirement for DO downregulation rather than upregulation when antigen presentation by B cells is in high demand in the pre-GC response (that is the gate-keeper for GC entry) and in the GC reaction^18–21^. However, it remains possible that, prior to down-regulation, DO association enhances the specific activity of DM enzymatic function in acidic MIIC through structural stabilization and reciprocal chaperoning, as suggested^12,36^.

Our single cell measurements showed the highest DM_free_/CLIP_freq_ in GC B cells (Fig. 6b), reflecting the importance of effective DM editing during B cell affinity maturation in GC. The noticeable presence of single cells with particularly low DM_free_/CLIP_freq_ appearing in the GC population from each tested donor probably reflect differentiated cells with significantly low to zero amounts of total DM (leading to low to zero amounts of DM_free_). This possibly reflects shortened DM half-life in the absence of DO chaperoning.

### Importance of tunable DO levels in memory B cells

Analyses using DM_free_/CLIP_freq_ revealed that local DM/DO stoichiometry, tuned by DO levels, controls the threshold of effective DM editing. The tuning function of DO likely leads to the significantly higher levels of CLIP that survive DM editing and are detectable in non-GC (DM/DO ratio^lo^) B cells compared to GC B cells (DM/DO ratio^hi-∞^), consistent with previous observations^19^.

The importance of DO tuning is further revealed by our single cell analysis of memory B cells. Memory B cells isolated from human tonsil tend to possess low DM_free_/CLIP_freq_, and high CLIP_LAMP1_/CLIP_tot_, despite lower CLIP_srf_ on memory compared to naïve cells^19,21^ and comparable levels of CLIP_tot_ between the two non-GC populations. The elevated percent of MIIC-localized CLIP/MHCII in memory B cells may result from both quiescent DM editing and BCR ligation-induced intracellular accumulation of CLIP/MHCII complexes during prior antigen exposure, as suggested^48^. The apparently quiescent level of DM editing is a result of limited DM activity free from DO inhibition/chaperoning in MIIC rather than insufficient amount of DM_tot_. Therefore, memory B cells are poised to be potent APC with more peptide-receptive MHCII and optimized active DM_free_ to exchange high affinity ligands efficiently upon antigen re-exposure. Our discovery that DO downregulation is synchronized with BCR affinity-dependent antigen internalization (within 20 min) supports our hypothesis that memory B cells expressing affinity matured BCRs rapidly potentiate DM editing by fine-tuning DM/DO stoichiometry. This response is faster than the time required for transcriptional upregulation of DM or DR that might also contribute to DM editing and peptide loading. The BCR affinity-dependent DO tuning provides insight into mechanisms to optimize memory B cells as APC and to their efficient recruitment into GC.

The important role of DO tuning is also observed in the upregulation of DO in memory B cells (vs GC B cells). It is a tempting idea that, within GCs, B cells (including GC-dependent memory B cells) with relatively lower affinity BCR lack sufficient antigen-internalization as a driver for continuous DO suppression. This could lead to their becoming memory B precursors that restore DO expression to control DM editing and allow bi-directional chaperoning. Another hypothetical model for DO restoration in extra-follicular memory B cells is that the daughter, memory-prone cells inherit the majority of DO molecules as a result of asymmetric division of activated B cells^49,50^ with the other daughter cell inheriting little due to its GC B cell fate. This is in line with our observation that there is a great amount of DO left even though DO level decreases during *in vitro* stimulation (see Fig. 4b).

### 2-synchronized-affinity-checkpoint model

Our investigation of DO function reveals its role in the synchrony of the aforementioned steps 1 and 2, involving affinity checks for ligand-receptor interaction along the antigen presentation pathway in B cells (Fig. 6e). The surface BCR affinity checkpoint at Step 1 sets a high bar for the specificity of antigen that is recognized and efficiently internalized by high affinity BCRs. The intracellular affinity checkpoint at Step 2 is synchronized with antigen internalization by DO down-regulation, which potentiates DM editing to ensure efficient peptide exchange for high affinity antigenic MHCII ligands. These ligands become locally available in MIIC likely due to the delivery and protection of antigen from degradation by high affinity BCR^51^ and the intersection of the intracellular trafficking paths of BCR-bound antigen and nascent MHCII^1,50^. Further, in findings that imply facilitated hand-off of Ig-associated antigen for MHCII binding, we have previously shown that cross-linking of surface BCR increases the subsequent co-precipitation of Ig and free DM^52^. As DO-associated DM does not co-precipitate with Ig, this is likely another benefit of synchronized antigen binding and DO downregulation.

Several lines of evidence imply potential mechanisms underlying rapidly responsive, BCR affinity-dependent DO tuning. Although no other study demonstrates a direct correlation between BCR affinity and downstream effects on regulation of the peptide loading machinery, it has been suggested that the receptor (including BCR) signaling on APCs can lead to acidification of late-lysosomal compartments^53,54^. Therefore, it is possible that high affinity BCR not only favors antigen internalization and protection of antigen from proteolysis before delivery into MIIC^51^, but also triggers signals that drive significant acidification of MIIC. The resultant acid-activated proteases ensure the generation of MHCII ligands from BCR-protected antigen and likely facilitate the destruction of acid-labile DO^12,30^. Indeed, the combined effects of acid and proteases appear to significantly accelerate DO reduction kinetics; in the results reported here, we observed a rapid change in DO levels in activated B cells (0.3–2 h), whereas our *in vitro* data on DO stability in acidic pH alone showed a timeframe of 1–3 days for DO denaturation^12^.

Because of this fast DO tuning, the established synchrony between antigen-BCR internalization and DM editing ensures quick reciprocal feedback during T-B interaction. This model applies to many situations when B cell antigen presentation and recruitment of T cell help is pivotal, especially at pre-GC and GC phases. In those phases, the fate of a B cell largely relies on BCR affinity and the surface density of antigenic peptide/MHCII complexes^2,3^, which is governed by intracellular DM editing. Our model predicts that antigen presentation by affinity-matured memory B cells is more effective for recruiting T_FH_ help and driving humoral immunity, as antigen internalization by high affinity BCR promptly potentiates DM editing. This peptide selection results in long-lasting surface peptide/MHCII complexes to support pre-GC interactions that seed and sustain GC reactions. This is particularly important at the time of initial B cell activation by antigen, when local cognate T cell help may not be immediately available.

Differences in DO alleles, albeit subtle, affect DO abundance and activity in humans and mice^44^, and peptidomes differ between DO^+^ and DO^null^ APCs^15,42,55^. As a result of these differences and variations in other parameters that guide B cell responses in different individuals, pre-GC activation of naïve/memory B cells and subsequent GC entry or GC-independent B cell activation may lead to significantly different immune responses that are either beneficial or detrimental (Fig. 6e). In the extreme, DO downregulation by DO-knockout or upregulation by DO ectopic expression may potentiate or attenuate DM editing, respectively, and lead to protective or deficient humoral responses or break self tolerance, as demonstrated in several mouse studies^44–46^. However, the basic goal of fine-tuning DM/DO stoichiometry in B cells is to facilitate coordination of antigen-BCR binding and binding of immunodominant peptides from that antigen to MHCII at the two synchronized affinity checkpoints. This system likely evolved to help maintain the immune balance between host defense and self-tolerance. Understanding these underlying mechanisms may inform future strategies to manage humoral immunity for therapeutic benefits.

## Methods

### Human B cell lines

T2 cells are class II-deficient human TxB hybrids that express neither MHCII nor DM/DO proteins^56^. T2 derived stable transfectants used in this study included T2DR4, T2DR4DM^57^ (both cell lines were gifts from Dr. Lisa Denzin, Rutgers Cancer Institute of New Jersey), and T2DR4DMDO cells^12^ (constructed by the Mellins labarotory). These cells were cultured in complete Iscove’s Modified Dulbecco’s Medium (IMDM) with GlutaMAX and 10% heat inactivated fetal bovine serum (FBS, Thermo Fisher Scientific). The expression of DR4, DM or DO in each cell line was maintained to an optimal level under the selection of appropriate antibiotics, as previously described^12^. DP cells are Epstein-Barr virus (EBV)-immortalized human B cell clones that secret anti-GAD65 autoantibodies: DPA or DPD^40,58^ (gifts from Dr. Anne-Marie Madec, INSERM U1060, Faculté de médecine Lyon-Sud, Oullins Cedex, France). The DP lines were cultured in complete IMDM with OPI media supplement (Millipore Sigma). All cell cultures in this study were maintained in a 37 °C incubator constantly supplied with 5% CO2.

### Human tonsil B cell isolation

Deidentified tonsil tissues were obtained under a Stanford Institutional Review Board approved protocol (IRB #38079). Tonsil tissues that were removed in a tonsillectomy procedure were minced to small sample pieces. These samples were washed with RPMI 1640 media (without supplements, Thermo Fisher Scientific) and passed through a 70 μm stainless steel cell strainer (BD Falcon) using a glass plunger to create a single cell suspension in 50 ml centrifuge tubes. Cells were then pelleted by centrifugation at 1500 rpm, room temperature (RT) for 5 min, resuspended in 30 ml RPMI 1640, and incubated at RT in a T150 Flask to remove tissue debris. Following 30–60 min of incubation, the cells were gently washed with RPMI 1640, passed through another 70 μm cell strainer into a new 50 ml centrifuge tube, and pelleted by centrifugation at 1500 rpm, RT for 5 min. The cell pellet was resuspended in 2–3 ml PBS + 5% heat inactivated FBS and added to a pre-washed nylon wool fiber column (Polysciences). The column with cells was incubated at 37 °C for 1 h, followed by 2–3 washes with PBS + 5% FBS to remove cells that were not attached to the nylon wool fiber (including T cells). Nylon wool adherent cells, the majority of which are B cells, were released by plunging and collected into a 50 ml centrifuge tube. After washes with PBS + 5% FBS and centrifugation at 1200 rpm for 10 min, cells were counted and resuspended in an appropriate amount of freezing media (RPMI-1640 containing 10% DMSO, 10% FBS, 1% penicillin/streptomycin, and 1% glutamine) for cryopreservation.

### Antibodies

Monoclonal antibodies (mAb): L243 (ref.^59^), Map.DM1 (ref.^60^), Mags.DO5 (ref.^19^), and CerCLIP.1 (ref.^57^) were used to specifically stain DR, DM, DO, and MHCII-associated CLIP, respectively. Fluorophore-conjugated forms of these mAbs used in this study included FITC-conjugated L243 (BioLegend), PE-conjugated anti-HLA-DR mAb (BD Biosciences), Alexa Fluor 568-conjugated L243, Alexa Fluor 647-conjugated L243, APC/Cy7-conjugated L243 (BioLegend), Alexa Fluor 488-conjugated Map.DM1, PE-conjugated Map.DM1 (BD Biosciences), Alexa Fluor 647-conjugated Map.DM1, Alexa Fluor 700-conjugaed Map.DM1, Alexa Fluor 568-conjugated Mags.DO5, CF405S-conjugated CerCLIP.1, FITC-conjugated CerCLIP.1 (BD Biosciences), Alexa Fluor 568-conjuaged CerCLIP.1, and Alexa Fluor 647-conjuaged anti-human CD74 (CerCLIP.1, Novus biologicals). Antibodies specific for endosomal markers used in 3D-SIM studies included Alexa Fluor 488-conjugated mouse anti-human CD107a (LAMP1) mAb (clone H4A3, Biolegend), Alexa Fluor 488-conjuaged mouse anti-human EEA1 mAb (clone 3C10, MBL), Alexa Fluor 488-conjugated polyclonal rabbit anti-human RAB7 (Bioss), polyclonal rabbit anti-EEA1 (Abcam), polyclonal rabbit anti-LAMP1 (Abcam), polyclonal rabbit anti-human RAB7 (a gift from Dr. Suzanne Pfeffer, Department of Biochemistry, Stanford), and Alexa Fluor 488-conjugated highly cross-adsorbed goat anti-rabbit IgG (Thermo Fisher Scientific). Antibodies used for the isolation and staining of tonsillar B cell subsets included FITC-conjugated anti-human CD3 (BD Biosciences), FITC-conjugated anti-human CD11c (clone Bu15, BioLegend), FITC-conjugated anti-human CD14 (clone M5E2, Biolegend), FITC-conjugated anti-human CD16 (clone 3G8, BioLegend), PerCP/Cy5.5-conjugated mouse anti-human CD19 (clone H1B19, BioLegend), PE/Cy7-conjugated anti-human IgD (clone IA6–2, BioLegend), Pacific Blue-conjugated anti-human IgD (clone IA6-2, BioLegend), Alexa Fluor 700-conjugated anti-human CD38 (clone HIT2, BioLegend), PE-conjugated anti-human CD69 (clone CH/4, Thermo Fisher Scientific), PE/CF594-conjugated mouse anti-human CD69 (clone FN50, BD Biosciences), PE/Cy7-conjugated anti-human CD86 (clone IT2.2, BioLegend), APC-conjugated anti-human CD86 (clone IT2.2, BioLenggend). Antibodies used in the study of BCR-GAD65 internalization included Alexa Fluor 488-conjugated goat anti-human IgG (Thermo Fisher Scientific), monoclonal mouse anti-GAD65 (Millipore Sigma), monoclonal mouse anti-GAD2, (ImmunoGen), and Alexa Fluor 488-conjugated highly cross-adsorbed goat anti-mouse IgG(H + L) (Thermo Fisher Scientific). To generate directly-conjugated CLIP-, DR-, DM-specific antibodies that are not commercial available, CerCLIP.1, L243, Map.DM1 were purified from mouse ascites using protein G beads (GE Healthcare), and then labeled with corresponding Alexa Fluor dye carboxylic acid, succinimidyl ester (Thermo Fisher Scientific), followed by size exclusion chromatography using a Superdex 200 gel filtration column (GE Healthcare) to remove extra free dye.

### Single cell cloning and culturing of T2-derived lines

3 million T2DR4DMDO cells were first labeled with LIVE/DEAD Fixable Dead Cell Stain (Thermo Fisher Scientific) in phosphate buffered saline (PBS) on ice for 30 min, and then washed and resuspended in 0.3 ml PBS + 1% bovine serum albumin (BSA) + 2 mM ethylenediaminetetraacetic acid (EDTA) for surface staining of DR4-associated CLIP. Cells were stained with FITC-CerCLIP.1 (1:5 dilution) on ice for 30 min and washed three times with 0.5 ml PBS + 1% BSA + 2 mM EDTA before resuspension in 0.5 ml PBS + 1% BSA + 2 mM EDTA for single cell sorting. Single cell fluorescence-activated cell sorting (FACS) was performed using a BD FACSAria II (BD Biosciences) at the Stanford Shared FACS Facility. Each cell (CLIP^hi^ or CLIP^lo^) was sorted into a well containing 100 μl complete IMDM with GlutaMax, 10% FBS, and 1% penicillin/streptomycin in a 96-well round bottom plate. Another 100 μl of complete IMDM GlutaMAX media were added into each well of the 96-well plate before the incubation of the plate at 37 °C. The top ~150 μl of media was carefully replaced with fresh media every 7 days until a clump of cells was observed at the bottom of the plate. Cells were then transferred to bigger flat-bottom wells of a 48-well plate with doubled or tripled volumes of fresh media + 1 mg/ml G418 (Geneticin) + 1 μg/ml puromycin + 100 μg/ml zeocin (Thermo Fisher Scientific) to maintain a selection pressure for the expression of DR4, DM and DO in sorted T2DR4DMDO clones. Under the appropriate selection condition, many single clonal lines maintained a consistent expression level of total DR4, DM or DO protein during several weeks of downstream scaling up and culturing. We also observed that the expression levels of these proteins could decrease if the selection reagent was removed from the culture.

### Isolation of tonsillar B cell subsets

Human tonsillar B cells were thawed on ice and washed twice with PBS + 5% FBS. The majority of dead cells and debris were separated from live B cells by density gradient centrifugation using Lymphocyte Separation Medium (MP Biomedicals). B cells were washed again, counted and resuspended in PBS at a density of 10×10^6^ cells/ml. Cells were stained with LIVE/DEAD Fixable Dead Cell Stain for 30 min. Following the live/dead staining, the cells were washed with PBS and resuspended in phenol red free RPMI (RPMI 1640 Medium, no glutamine, no phenol red, Thermo Fisher Scientific) supplemented with 3% FBS. Subsequently, the B cells were stained on ice with a cocktail of antibodies including: anti-CD3, anti-CD11c, anti-CD14, anti-CD16, anti-CD19, anti-CD38, and anti-IgD antibodies. After 30–40 min of surface staining, cells were washed in phenol red free RPMI and passed through a 70 μm filter before loading onto the cell sorter. FACS was performed using a FACSAria II at the Stanford Shared FACS Facility using a 70 μM nozzle. B cells (CD3−, CD14−, CD16−, CD11c−, CD19+) were sorted into naïve (IgD+, CD38−), memory (IgD−, CD38−) and GC (IgD−, CD38+) cell sub-populations, as previously described^18,19^. Sorted cells, resuspended in RPMI supplemented with 3% FBS, were kept on ice. Purity of sorted B cell subsets was confirmed by reapplying a sample onto the cell sorter right after sorting.

### Activation of tonsillar B cell subsets

Naïve, memory and GC B cells were incubated at a density of 10^6^ cells/ml with or without an activation cocktail in IMDM medium supplemented with 10% FBS, 1% Penicillin/Streptomycin, and 1% glutamine at 37 °C for various time periods prior to analysis by flow cytometry to determine total protein levels, by iFACS to calculate DM_free_/CLIP_freq_, or by 3D-SIM to quantify protein co-localization. The activation cocktail contained 20 ng/ml of IL-4 (Peprotech), 20 ng/ml of IL-2 (BioLegend), 200 ng/ml of MEGACD40L (human CD4 ligand, Enzo Biochem Inc), 5 μg/ml of Goat F(ab’)2 anti-human IgG-UNLB mouse adsorbed and 5 μg/ml Goat F(ab’)2 anti-human IgM-UNLB (Southern Biotech).

### Crosslinking of BCR on DP cell lines

DPA or DPD cells were incubated at a density of 2 × 10^6^ cells/ml with or without 5 μg/ml of Goat F(ab’)2 anti-human IgG in complete IMDM GlutaMAX medium supplemented with 10% FBS, 1% penicillin/streptomycin, and 1% glutamine at 37 °C for an indicated time length. At different time point (i.e., 0, 20 min, 1 h, and 2 h), 0.2 million cells were collected by centrifugation at 400 x g, 4 °C for 5 min and incubated at a density of 10 × 10^6^ cells/ml with 10 μg/ml Alexa Fluor 488-conjugated goat anti-human IgG in PBS + 1% BSA on ice for 30 min to stain surface BCR. After two washes with PBS + 1% BSA, cells were analyzed on a FACSCalibur flow cytometer (BD Biosciences).

### Stimulation of DP cell lines by GAD65

DPA or DPD cells were incubated at a density of 10 × 10^6^ cells/ml with or without 2 μg/ml GAD65 (Abcam) in PBS + 1% BSA on ice for 1 h to load GAD65 onto BCR while BCR internalization is inhibited. GAD65-loaded or non-loaded cells were washed with PBS + 1% BSA, pelleted by centrifugation at 400 x g, 4 °C for 5 min, resuspended at a density of 2 × 10^6^ cells/ml in complete IMDM GlutaMAX medium containing 5μg/ml GAD65 or no antigen, and then incubated at 37 °C for an indicated time length. At 0, 20 min, 1 h, and 2 h, 0.6 million cells were collected by centrifugation at 400 x g, 4 °C for 5 min and used in three different assays: 1) 0.2 million cells were used for iFACS analysis (see below). 2) 0.2 million cells were incubated at a density of 10 × 10^6^ cells/ml with 10 μg/ml Alexa Fluor 488-conjugated goat anti-human IgG in PBS + 1% BSA on ice for 30 min to stain surface BCR. 3) 0.2 million cells were incubated at a density of 10 × 10^6^ cells/ml with 10 μg/ml anti-GAD65 antibody on ice for 30 min to stain surface GAD65, followed by two washes with PBS + 1% BSA and the secondary staining with 10 μg/ml Alexa Fluor 488-conjugated goat anti-mouse IgG(H + L) in PBS + 1% BSA on ice for 30 min. Antibody-labeled cells in 2) and 3) were analyzed on a BD FACSCalibur flow cytometer.

### Bulk analysis of total MHC proteins by flow cytometry

Cells were fixed/permeabilized using the Cytofix/Cytoperm reagent (100 µl per million cells, BD Pharmingen) at RT for 20 min, and washed twice with 1x Permwash buffer (BD Pharmingen) prior to immunofluorescent antibody labeling. Washed cells were pelleted by centrifugation at 400 × g, 4 °C for 5 min and resuspended in 1x Permwash buffer containing fluorophore-conjugated DR-, DM- DO-, and CLIP-specific antibodies (~1 µg of each antibody per million cells per 100 µl buffer) and incubated on ice for 1 h. Antibody-labeled cells were washed twice with 1x Permwash buffer and then resuspended in PBS + 1% BSA for analysis on a flow cytometer, either FACSCalibur or LSRII (BD Biosciences). Flow cytometric data were analyzed using FlowJo software (Tree Star, Inc.).

### Determination of intracellular CLIP levels by flow cytometry

Cells were first labeled with LIVE/DEAD Fixable Aqua Dead Cell Stain in PBS on ice for 30 min, and then washed with PBS and stained with FITC-CerCLIP.1 (1:5 dilution) in PBS + 1% BSA at a cell density of 10×10^6^ cells/ml on ice for 30 min. After the incubation, cells were washed twice with PBS + 1% BSA and fixed/permeabilized as described above. Half of the fixed/permeabilized cells were resuspended in PBS + 1% BSA for flow cytometric analysis of surface CLIP levels; the other half was resuspended at a density of 10×10^6^ cells/ml in 1x Permwash buffer containing FITC-CerCLIP.1 (1:5 dilution) and incubated on ice for 30 min to stain intracellular CLIP. The second half of CLIP-labeled cells were further washed twice with 1x Permwash buffer and then resuspended in PBS + 1% BSA for flow cytometric analysis of total CLIP levels. The levels of intracellular CLIP that are associated with MHCII were calculated as,

$${\rm{MFI}}\,({{\rm{CLIP}}}_{{\rm{int}}})={\rm{MFI}}\,({{\rm{CLIP}}}_{{\rm{tot}}})-{\rm{MFI}}\,({{\rm{CLIP}}}_{{\rm{srf}}}).$$

### Index single-cell fluorescence-activated cell sorting (iFACS)

Cells were first labeled with LIVE/DEAD Fixable Aqua Dead Cell Stain in PBS on ice for 30 min, and then fixed/permeabilized and labeled with fluorophore-conjugated DR-, DM- DO-, and CLIP-specific antibodies using the same procedure as in the bulk analysis of total MHC proteins. BD FACSAria II cell sorter was used to sort single cells into a 96-well plate. During cell sorting, the index feature^61^ was enabled to record index fluorescence intensities (FIs). Index FIs included FI at each protein detection channel for individually sorted single cells. iFACS was performed at the Stanford Shared FACS Facility. Index FI data were exported in Excel from the BD FACSDIVA software (BD Biosciences) and can be analyzed separately from the Flow Cytometry Standard (FCS) files.

### Calculation of DM_free_/CLIP_freq_ using index FI data

CLIP removal for peptide exchange in B cell is largely dependent on DM catalytic function. Therefore, the relative amount of free DM molecules to MHCII-associated CLIP in each individual cell is a critical parameter that reflects the efficacy of CLIP removal. The index FI data associated with individually sorted single cells enables the calculation of such a parameter: DM_free_/CLIP_freq_. The higher the number is, the more effectively a cell can scan CLIP-loaded MHCII and catalyze CLIP release. As opposed to the tight DM/DO interaction^12,23,24^, DM only transiently associates with a peptide-receptive form of MHCII^5,6,62^. Therefore, DM molecules that are not DO-associated are free of substrate and capable of accessing CLIP-loaded MHCII and catalyzing peptide exchange. With the index FI data acquired for single cells, free DM can be calculated as:

$${{\rm{DM}}}_{{\rm{free}}}={{\rm{DM}}}_{{\rm{tot}}}-{{\rm{DM}}}_{{\rm{DO}}-{\rm{associated}}}$$

where DM_tot_ per cell was determined by index FI at the DM detection channel after normalization for cell cell (divided by FSC). DO-associated DM per cell was estimated by the index FI at the DO detection channel after normalization for cell size. The frequency of CLIP-loaded MHCII among all MHCII molecules within the same cell can be calculated as,

$${{\rm{CLIP}}}_{{\rm{freq}}}={{\rm{CLIP}}}_{{\rm{tot}}}/{{\rm{MHCII}}}_{{\rm{tot}}}$$

where CLIP_tot_ per cell was determined by index FI at the CLIP detection channel after normalization for cell size. MHCII_tot_ per cell was estimated by the index FI at the DR detection channel after normalization for cell size. Notably, because the comparison of DM_free_/CLIP_freq_ was performed among single cells from the same individual whose DR expression levels are most likely proportional to their MHCII expression levels; the usage of DR_tot_ (≈cMHC_tot_, where c is a constant) in the calculation of CLIP_freq_ should have minimal effect on these comparisons. Examples of these comparisons included tonsillar naïve vs memory B cells from the same donor, and DPA vs DPD monoclonal B cell lines isolated from the same patient who had developed type 1 diabetes^58^. In addition, as the index FIs representing DM and DO levels were measured by two independent detection channels in an iFACS experiment, it is likely that (FI_DM_/FSC – FI_DO_/FSC) for certain cells results in a negative value. However, for relative values, this has no influence on a comparison of efficacy in the catalysis of CLIP removal or peptide exchange between two groups of cells. For example, as compared to a negative value, a positive value means relatively more DM_free_ per cell. However, in order to match the physiological meaning of DM_free_, all (DM_tot_ − DM_DO-associated_) values in an experiment were subtracted by the minimum value of (DM_tot_ − DM_DO-associated_) from the same experiment twice (a linear transformation of the data) to adjust all values to positive with the minimum now becoming |DM_tot_ − DM_DO-associated_|_min_.

### Sample preparation for 3D-SIM

Cells were fixed/permeabilized using the Cytofix/Cytoperm reagent (BD Pharmingen) at RT for 20 min (for cell lines) or on ice for 30 min (for tonsillar B cells) and washed twice with 1x Permwash buffer (BD Pharmingen). For FACS-sorted tonsillar B cells (before or after *in vitro* activation), fixed/permeabilized cells were exposed to the high intensity white light from the Metal Halide Light Source (MME-250, 250 W type, TRI Vision, www.triv.co.kr) for 2 h on ice to completely photo bleach residual fluorophores (validated by flow cytometry and 3D-SIM). Fixed/permeabilized cells were then stained with antibodies specific for an endosomal marker and selected proteins in 1x Permwash buffer + 50 mM glycine using the same approach as used above for flow cytometric analysis. The excess of antibodies in the staining solution were depleted by extensive washes: 2–3 washes using 1x Permwash + 50 mM glycine followed by 2–3 washes using PBS + 1% BSA + 50 mM glycine. Glycine was added to inhibit non-specific binding. Alex Fluor dyes were used due to their brightness and photostability. There was minimal difference when detecting endosomal localization of MHC proteins by a direct staining (i.e., using Alexa Fluor dye directly conjugated LAMP1, EEA1 or RAB7) versus an indirect staining (i.e., using rabbit anti- LAMP1, EEA1 or RAB7, followed by secondary Alexa Fluor dye conjugated anti-rabbit antibodies). In experiments that generated data for quantification of surface-surface co-localization, the direct staining was selected to minimize signal to noise ratios. Alexa Fluor 488 was used for endosomal markers, Alexa Fluor 568 was used for CLIP or DO, and Alexa Fluor 647 was used for DR or DM. Antibody-labeled cells were resuspended in PBS + 1% BSA + 50 mM glycine at a density of 10^6^ cells/ml and 100 μl transferred to an empty Shandon single cytofunnel with white filter cards (Thermo Scientific Fisher) that had been pre-treated by washing with 1 ml PBS followed by cytospinning. The funnel with cell samples was then assembled with a high precision cover glass (24 × 50 mm, 170 ± 5 μm, No. 1.5 H, MARIENFELD) and a Shandon cytology stainless steel slide clip (Thermo Scientific Fisher) to cytospin the cells onto the coverslip and form a monolayer cell pellet. The cytospin was performed using a cytocentrifuge at slow acceleration mode 500 rpm for 5 min. After disassembling, the coverslip was air-dried briefly and the spot where the monolayer cell pellet formed was covered by 14 μl of SlowFade Gold Antifade mountant with DAPI (Thermo Fisher Scientific). The coverslip was then inverted and mounted over a pre-cleaned Gold Seal plain micro slide (25 × 75 mm, Thermo Fisher Scientific) and the edge was sealed by nail polish.

### 3D-SIM data acquisition and reconstruction

Applied Precision N = 1.514 immersion oil (GE Healthcare) was applied on the coverslip. The micro slide was inverted and placed in position over the objective lens (U-PLANAPO 100×, N.A. 1.40) on the OMX V4 microscope platform with the sample and coverslip side facing down. Images were acquired at SIM mode using Applied Precision DeltaVision OMX imaging system (GE Healthcare) with four lasers (100 mW: 405 nm, 488 nm, 568 nm; 300 mW 642 nm MONET) and 3 emCCD detection cameras. The wavelengths for emission filters are 435/31 nm for the DAPI or equivalent dye channel, 528/48 nm for the Alexa Fluor 488 or equivalent dye channel, 609/37 nm for the Alexa Fluor 568 or equivalent dye channel, and 683/40 nm for the Alex Fluor 647 or equivalent dye channel (detection using the same camera as for the DAPI channel). Signal (maximum) to noise (minimum) ratio at each channel was optimized (>10 fold), and the laser power (%T) was set to ≤10% and exposure below ≤80 ms to minimize photobleaching. The size of T2 and tonsillar B cells are ~5–10 μm and ~2–5 μm, respectively. A 512 × 512 pixels (0.08 μm/pixel) XY microscopic field of interest, or more accurately, a 3D space in between the coverslip and the micro slide that contains the monolayer cell specimen was scanned along Z-axis at 0.125 μm steps. ~50–80 scanned optical sections of the 3D space containing the specimen yielded a stack of ~50–80 2D XY images. The stack of images was then reconstructed into a volumetric 3D image using softWoRx 7.0.0 image processing software (GE Healthcare). OMX structured illumination (SI) reconstruction was performed using channel-specific optical transfer function (OTF) with channel-specific wiener filters: 0.0030 for the DAPI channel, 0.0010 for the Alexa Fluor 488 channel, 0.0010 for the Alexa Fluor 568 channel, and 0.003 for the Alex Fluor 647 channel. OMX images were corrected for chromatic aberration and aligned using the blue-green-red (BGR) image source drawer.

### Calculation of surface-surface co-localization using 3D-SIM data

The SI reconstructed 3D image was viewed and analyzed using Imaris v9.4 image analysis software (ImarisXT, Bitplane Inc, http://bitplane.com). For each source channel, a volumetric surface connecting voxels (0.08 um x 0.08 μm x 0.08 μm) that contain fluorescence signals associated with the target protein was generated to simulate the localization of the protein. To minimize noise, the algorithm allowed smoothing by 0.08 μm “Surfaces Area Detail Level” and background subtraction by 0.16 μm “Diameter of largest Sphere that fits into the Object”. The filter used to classify surface was “Number of Voxel above” 5. With volumetric surfaces available at two source channels, an overlapping surface was then calculated using the “surface-surface colocalization” Xtension, a METLAB-based algorithm built as an extension patch for Imaris. This Xtension generated a new channel for the overlapping surface that includes voxels shared by the two source channel surfaces. The volumetric summation of voxels within the overlapping surface divided by the volumetric summation of voxels within one surface at a source channel was used to estimate the percent of the protein represented by the source channel that is co-localized with the other protein.

### Statistical analysis

All statistical analyses in this study compared a single variable between two groups of samples. Therefore, a two-tailed *t*-test was used to determine the statistical significance of the difference, with significance defined as P < 0.05. Welch’s t-test was used if the two groups of samples had unequal variances or unequal sample sizes. The paired samples t-test was used if there were matched pairs of samples in the two groups. All statistics were performed with GraphPad Prism, using the built-in analysis.

## Supplementary information

Supplementary Info

Supplementary Movie 1

Supplementary Movie 2

Supplementary Movie 3

Supplementary Movie 4

Supplementary Movie 5

Supplementary Movie 6

Supplementary Movie 7

Supplementary Movie 8

Supplementary Movie 9

Supplementary Movie 10
