# Supplementary material for: Synergy between B cell receptor/antigen uptake and MHCII peptide editing relies on HLA-DO tuning
Source: Sci Rep. 2019 Sep 25;9:13877. doi: 10.1038/s41598-019-50455-y (PMC6761166; doi:10.1038/s41598-019-50455-y)
Supplement: Supplementary file 1 — Supplementary Info [file 41598_2019_50455_MOESM1_ESM.docx]

**Supplementary information**

**Synergy between B cell receptor/antigen uptake and MHCII peptide editing relies on HLA-DO tuning**

Wei Jiang^1,2,^*, Lital N. Adler^1,2,3^, Henriette Macmillan^1,2,4^, Elizabeth D. Mellins^1,2,^*

**Affiliations:**

^1^Department of Pediatrics – Human Gene Therapy, Stanford University School of medicine, Stanford, CA 94305, USA

^2^Stanford Immunology, Stanford University School of Medicine, Stanford, CA 94305, USA

^3^Current address: Weizmann Institute of Science, Rehovot Area, Israel

^4^Current address: Department of Medicine, University of California San Francisco, San Francisco, CA 94143, USA

*Correspondence to: [mellins@stanford.edu](mailto:mellins@stanford.edu) or [wjiang6@stanford.edu](mailto:wjiang6@stanford.edu)

This document includes 7 supplementary figures and the caption for 10 supplementary movies.

**Supplementary figures**

**
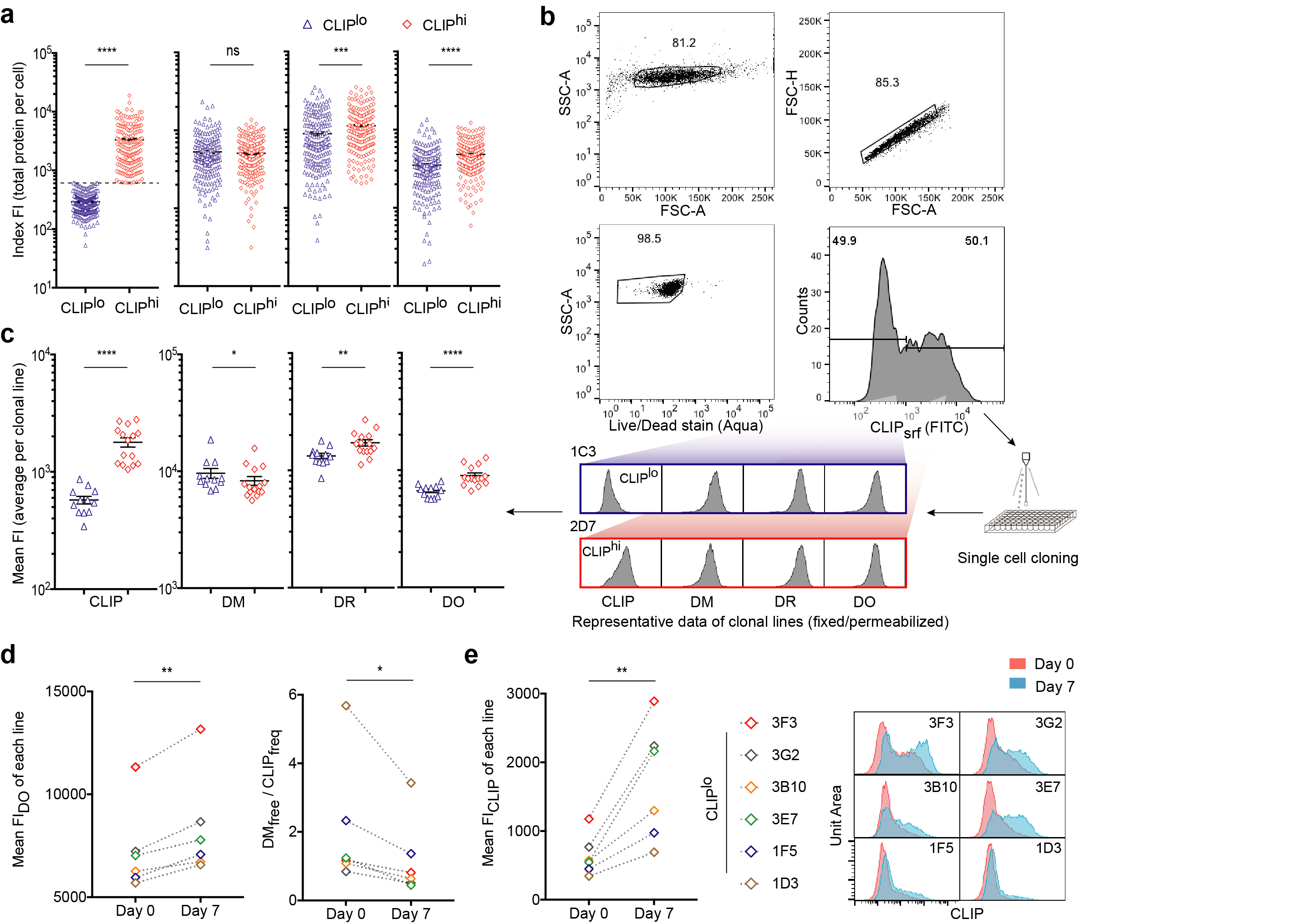
**

**Supplementary Fig. 1** **Single-cell measurements for calculation of DM_free_/CLIP_freq_. a**, Comparisons of average index FIs between CLIP^lo^ and CLIP^hi^ groups, based on an arbitrary separation as indicated by the dotted line. T2DR4DMDO cells were analyzed by iFACS, as in **Fig. 1b**. **b**, Illustration of gating strategies for FACS sorting of live single T2DR4DMDO cells after surface staining of CLIP. Histograms at the bottom show the flow cytometric analysis of two representative single clonal lines, 1C3 from the CLIP^lo^ group and 2D7 from the CLIP^hi^ group. These clonal lines were fixed/permeabilized, co-stained for DM, DO, DR, CLIP, and analyzed by flow cytometry. **c**, Comparison of mean FIs representing average target protein level per clonal line between CLIP^lo^ and CLIP^hi^ groups. Single T2DR4DMDO clonal lines were fixed/permeabilized and analyzed by flow cytometry as in (**b**). **d**, Pairwise comparisons (paired *t*-test) between mean FI_DO_ (left) or DM_free_/CLIP_freq_ (right) of each line before (Day 0) and after (Day 7) upregulation of DO expression. **e**, Left panel: A pairwise comparison (paired *t*-test) between mean FI_CLIP_ of each line before (Day 0) and after (Day 7) the upregulation of DO expression. Right panel: overlay of histograms showing the CLIP levels of each line before (Day 0) or after (Day 7) upregulation of DO expression. ns: non-significant, p>0.05; *p<0.05, **p<0.01, ****p<0.0001.


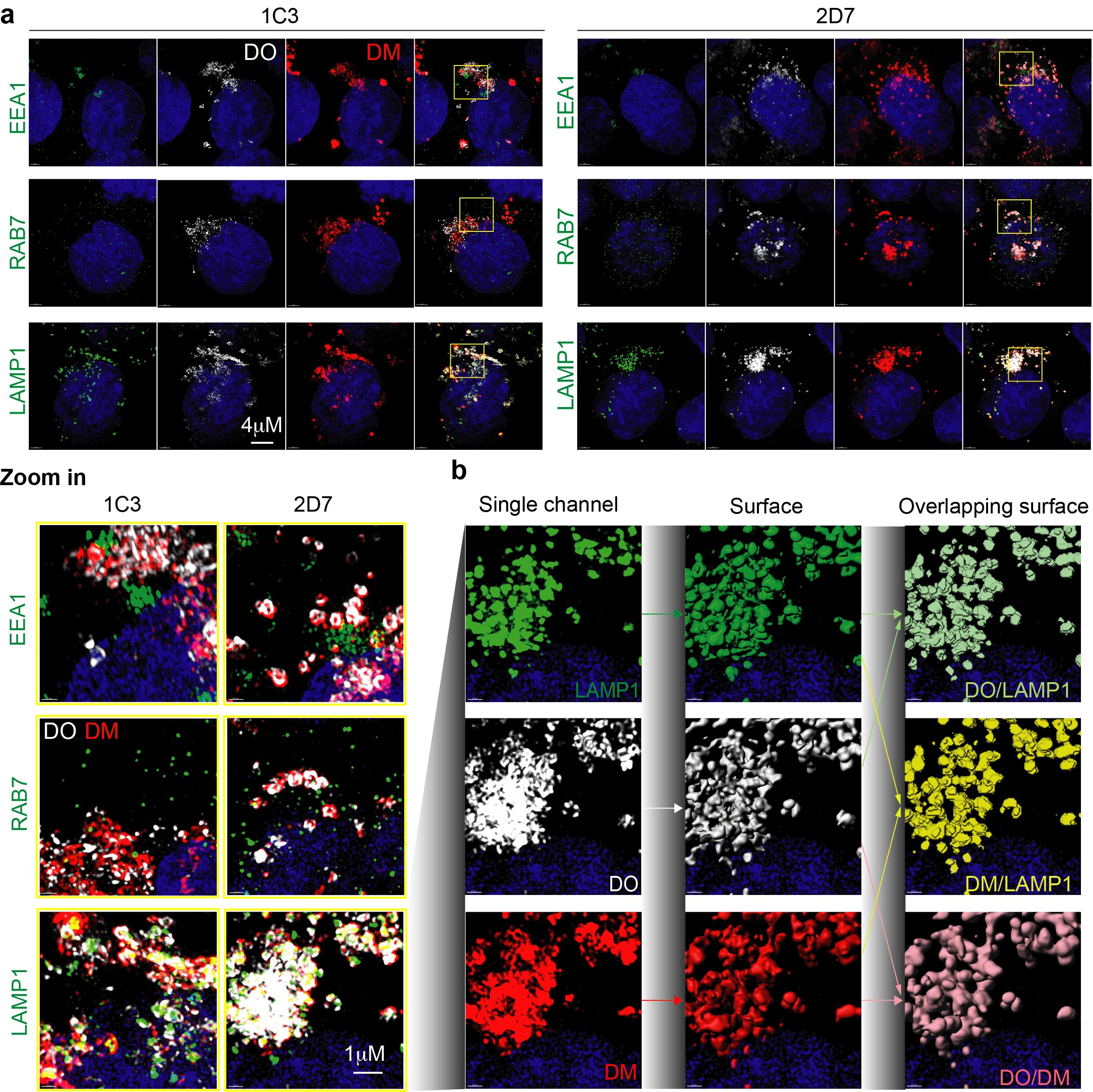


**Supplementary Fig. 2 Super-resolution measurement for co-localization by 3D-SIM. a**, Representative 3D-SIM single channel or overlay views of fixed/permeabilized 1C3 or 2D7 co-stained for DM (red), DO (white) and the indicated endosomal marker (green). Localization of EEA1 and RAB7 or co-localization of LAMP1 with DM and DO are revealed in high-resolution zoom-in views (lower). **b**, Quantification of co-localization. A volumetric surface connecting voxels that contain fluorescence signals in each channel was first generated, and then the overlapping surface connecting voxels shared by two surfaces was calculated. The summation of voxels within the overlapping surface divided by the summation of voxels within the single channel surface results in the best estimation of the percent of one protein that is co-localized with another.


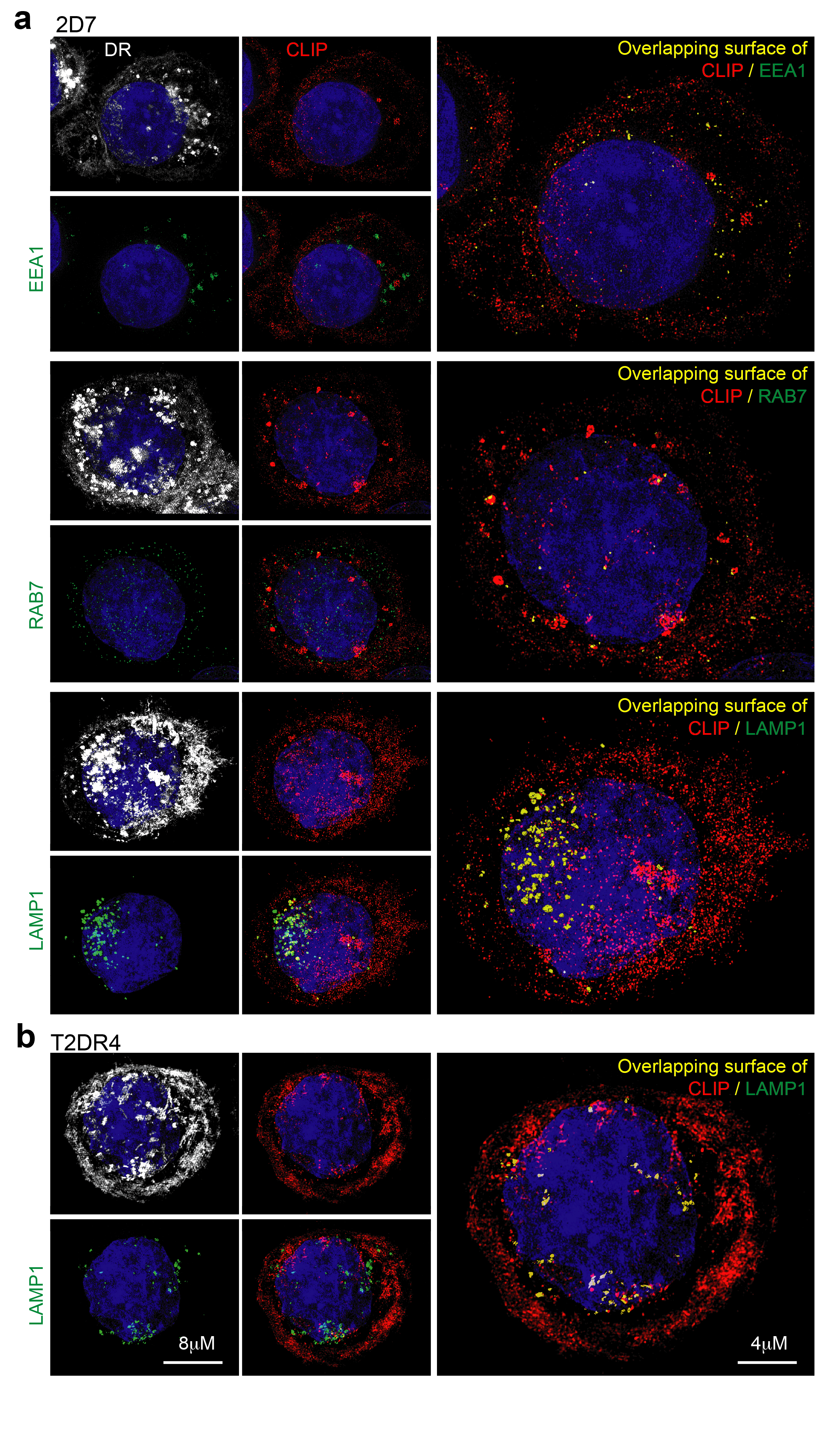


**Supplementary Fig. 3 Super-resolution quantification distinguishes CLIP_int_ (CLIP_LAMP1_) from CLIP_srf_. a**, Representative 3D-SIM single channel or overlay views of fixed/permeabilized 2D7 co-stained for DR (white), CLIP (red), and the indicated endosomal marker (green). The image to the right shows an overlay view of the CLIP channel and the calculated endosomal marker/CLIP overlapping surface (yellow). **b**, Representative 3D-SIM views as in (A), but of fixed/permeabilized T2DR4 co-stained for DR (white), CLIP (red), and LAMP1 (green).


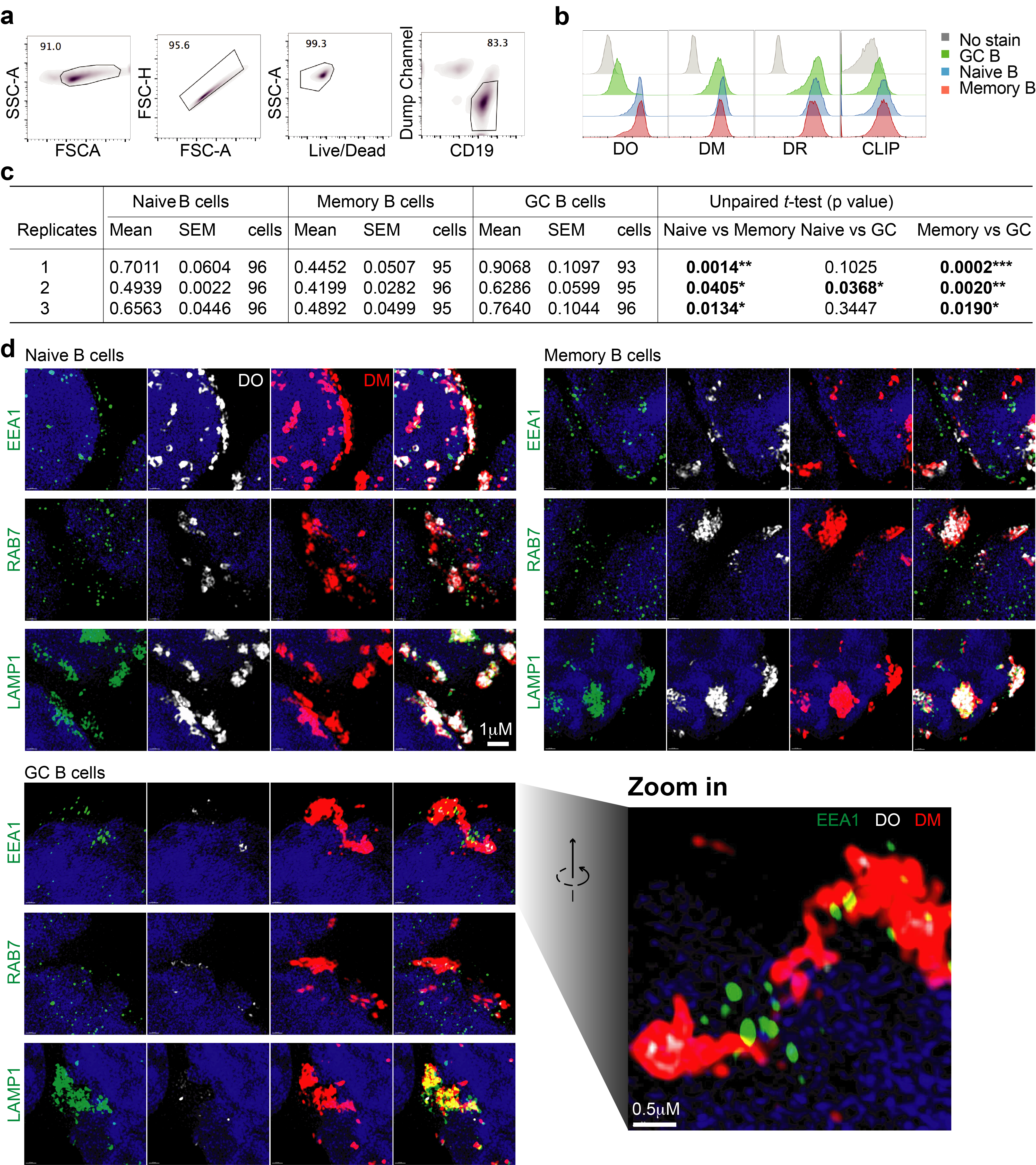


**Supplementary Fig. 4 Low DM_free_/CLIP_freq_ in memory B cells. a**, Illustration of gating strategies for FACS of live human tonsillar B cells that were sorted into naïve, memory and GC sub-populations as shown in **Fig. 3a**. Surface markers in the dump channel are CD3, CD11c, and CD16. **b**, Representative histograms showing levels of the indicated proteins in B cell subpopulations. **c**, A comparison of the mean of DM_free_/CLIP_freq_ calculated from individual cells among different B cell subpopulations. Significant differences are bolded: *p<0.05, **p<0.01; ***p<0.001. **d**, Representative 3D-SIM single channel or overlay views of fixed/permeabilized B cells co-stained for DM (red), DO (white), and the indicated endosomal marker (green). An enlarged 180**°** rotated view reveals the spatial separation of DM from most of EEA1 in GC B cells.


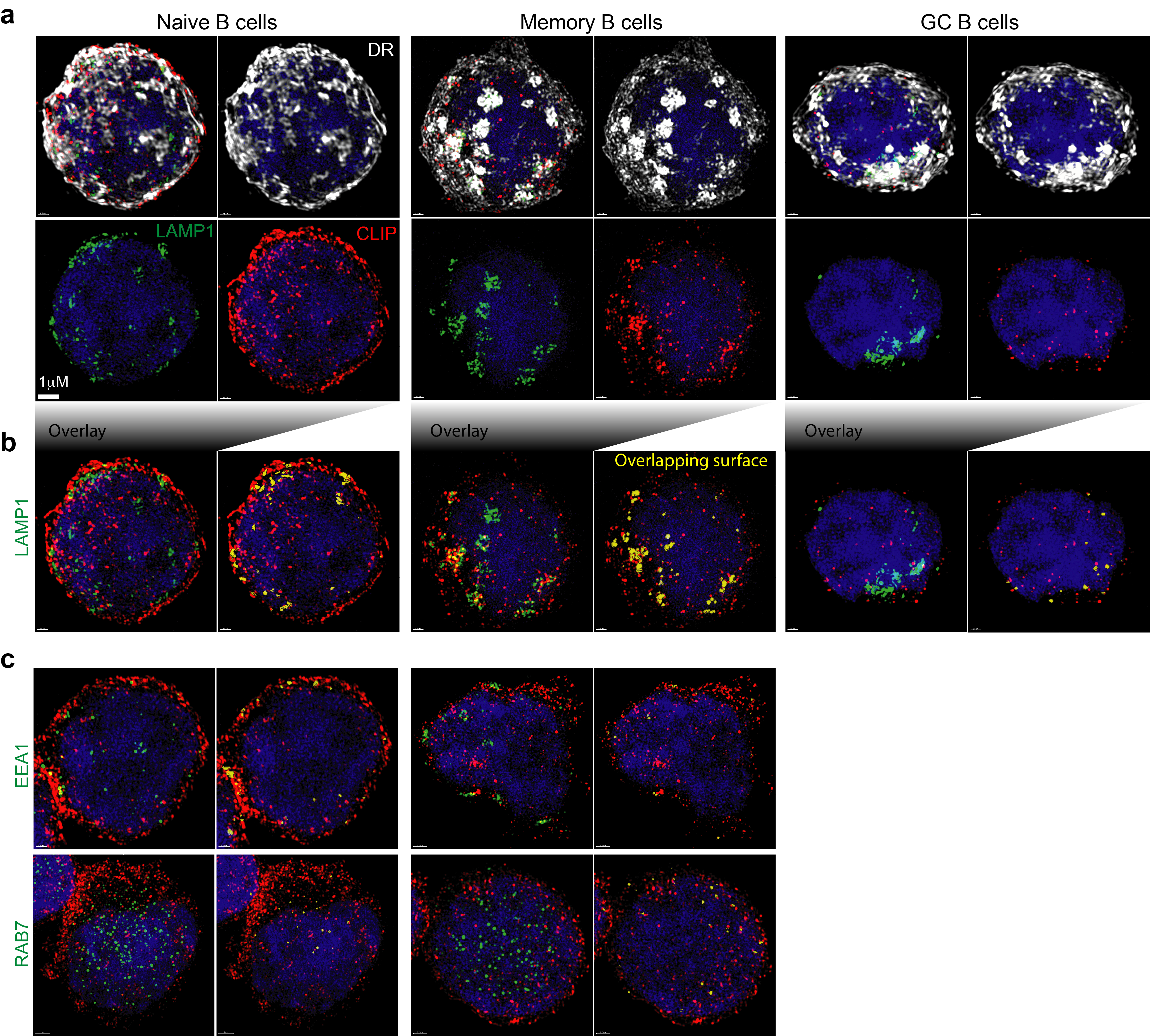


**Supplementary Fig. 5 High %CLIP co-localizes with LAMP1 in memory B cells. a**, Representative 3D-SIM overlay and single channel views of fixed/permeabilized B cells co-stained for DR (white), CLIP (red), and LAMP1 (green). **b**, The overlay view of the CLIP channel (red) with the LAMP1 channel (green) or with the calculated LAMP1/CLIP overlapping surface (yellow). **c**, The overlay view of the CLIP channel (red) with the indicated endosomal marker channel (green) or with the calculated endosomal marker/CLIP overlapping surface (yellow).


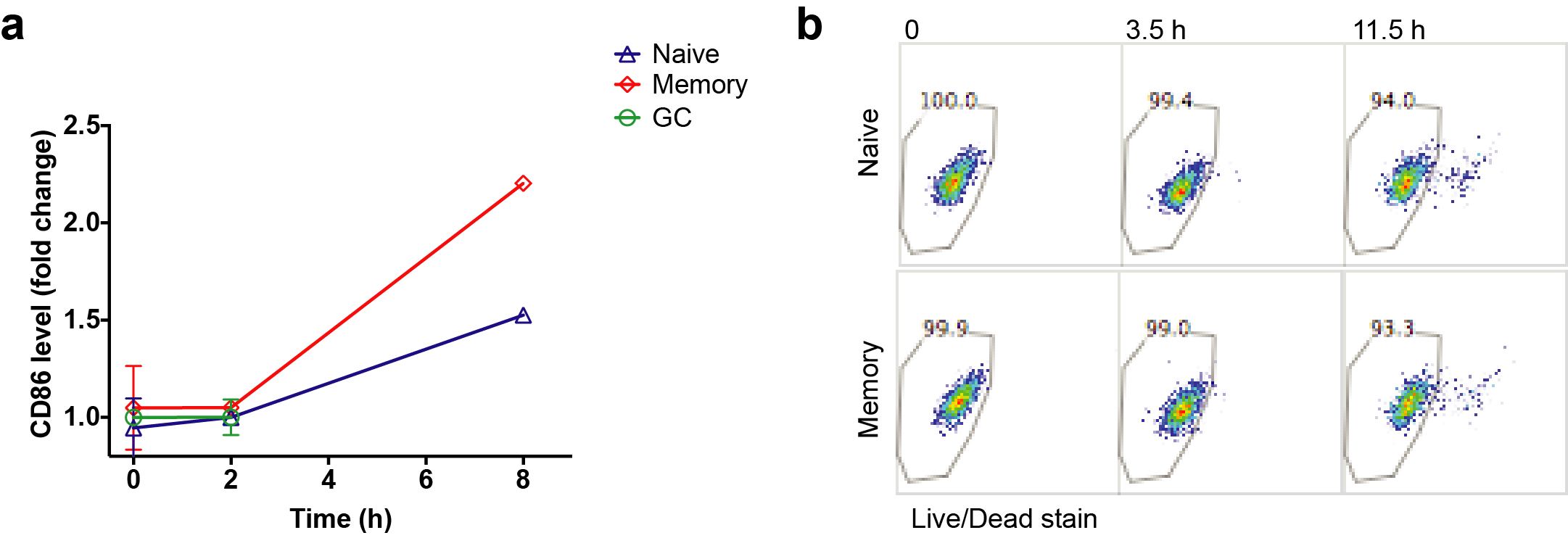


**Supplementary Fig. 6** ***In vitro* activation of human tonsil B cells while limiting cell death. a**, Different subtypes of tonsil B cells were stimulated as in **Fig. 4a** for the indicated time, fixed/permeabilized before staining for total CD86, and then analyzed by flow cytometry. MFI fold change of stimulated over unstimulated samples was normalized to that of GC B cells at time 0. Data are represented as mean±SEM. n=3. **b**, Flow cytometric analysis of cells cultured and stimulated at the condition used for the *in vitro* activation showing increased numbers of dead cells that take-up more Live/Dead staining dye after 3.5 h. Gates indicate live cells.

**
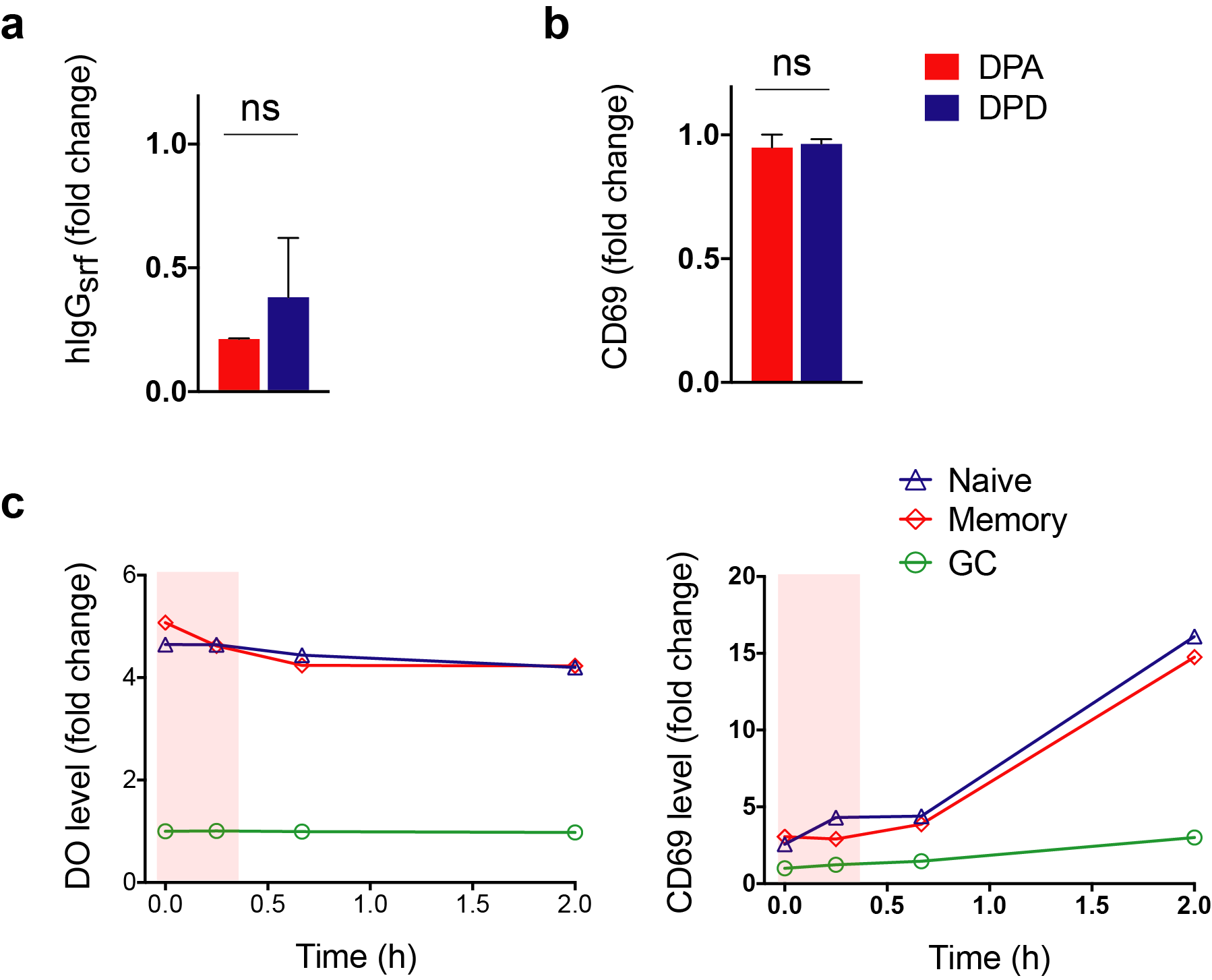
**

**Supplementary Fig. 7** **Fast DO downregulation, whereas CD69 level is unchanged. a**, Internalization of surface human IgG (fold change<1) in both DPA and DPD cells after incubation with anti-hIgG antibodies at 37°C for 20 min. Data are represented as mean±SEM. ns: no significance, p>0.05. n=3. **b**, Unchanged total CD69 levels in GAD65-loaded DPA and DPD cells that were further incubated with GAD65 at 37°C for 20 min and fixed/permeabilized before flow cytometric analysis. Data are represented as mean±SEM. ns: no significance, p>0.05. n=5. **c**, Negligible CD69 upregulation in memory B cells within 20 min *in vitro* stimulation. Different subtypes of tonsil B cells were stimulated as in **Fig. 4a,** for the indicated time, fixed/permeabilized before co-staining of total DO and CD69, and then analyzed by flow cytometry. MFI fold change of stimulated over unstimulated samples was normalized to that of GC B cells at time 0.

Captions for Supplementary Movies 1-10

**Movie 1. 2D7 cells co-stained for DM (red), DO (white) and LAMP1 (green)**

**Movie 2. 1C3 cells co-stained for DM (red), DO (white) and LAMP1 (green)**

**Movie 3. 2D7 cells co-stained for CLIP (red) and LAMP1 (green)**

**Movie 4. 2D7 cells co-stained for CLIP (red) and EEA1 (green)**

**Movie 5. 2D7 cells co-stained for CLIP (red) and RAB7 (green)**

**Movie 6. Naive B cells co-stained for DM (red), DO (white) and LAMP1 (green)**

**Movie 7. Memory B cells co-stained for DM (red), DO (white) and LAMP1 (green)**

**Movie 8. GC B cells co-stained for DM (red), DO (white) and LAMP1 (green)**

**Movie 9. Naive B cells co-stained for CLIP (red) and EEA1 (green)**

**Movie 10. Memory B cells co-stained for CLIP (red) and EEA1 (green)**
